# Supplementary material for: Health octo tool matches personalized health with rate of aging
Source: Nat Commun. 2025 May 5;16:4007. doi: 10.1038/s41467-025-58819-x (PMC12053696; doi:10.1038/s41467-025-58819-x)
Supplement: Supplementary file 1 — Supplementary Information [file 41467_2025_58819_MOESM1_ESM.pdf]

Supplementary Materials.

Study Populations

BLSA: The Baltimore Longitudinal Study of Aging (BLSA) is a cohort study focusing on healthy aging in community-dwelling participants. Information on physical examinations, laboratory tests, and medical records was extracted from this study[1]. Participants aged ≥20 years were originally recruited, and their follow-up intervals were as follows: quadrennial for individuals younger than 60, biennial for those aged 60–79, and annual for those aged ≥80 or with any disease at any age. The analysis included 456 men and 451 women (total N=907) with three subsequent visits, spanning dynamic intervals with a mean of 3.9 ± 2.9 SD years and a follow-up duration ranging from 1 to 13 years after the last visit, continuing through 2019. Mortality follow-up was carried out for an average of 3.1 ± 2.3 years.

InCHIANTI: The Invecchiare in Chianti (InCHIANTI) study is a prospective population-based study of older individuals in Tuscany, Italy [2]. We included 986 participants aged 21 to 98 years (435 men and 551 women) with a follow-up duration of 6 years.

NHANES: For additional evidence and replication, we utilized National Health and Nutrition Examination Survey (NHANES) data, which covered the years 2003 to 2018. This dataset comprised 44,790 individuals aged 18 years and older, with 23,121 women. Of note, in the NHANES database individuals older than 85 years were coded as 85 years [3].

Table S1: Body Organs and Their Corresponding Disease Level Definitions Based on Disease Severity or level.

**Cardiovascular System (CV)**	
Hypertension (HTN; 1-3)	1: No HTN<br>2: Systolic Blood Pressure (SBP)>140; Diastolic Blood Pressure (DBP)>90 or documented history of HTN and no medication<br>3: SBP>140; DBP>90 or documented history of HTN and medication<br>HTN: medication (hydropyridines calcium channel blocker (CCB), diuretics, angiotensin converting enzyme inhibitors, beta blockers, and other antihypertensive drugs).
Ischemic Heart Disease (IHD; 1-3)	1: No IHD<br>2: History of prolonged angina pectoris (more than 5 minutes), history of angioplasty, or coronary artery bypass surgery (CABG) 3: Myocardial infarction: prolonged chest pain: 300 minutes or more, significant q wave on EKG, documented history of myocardial infarction, or nitrate medication.
Congestive Heart Failure (CHF; 1-3)	1: No CHF<br>2: CHF with preserved ejection fraction (EF) in echocardigraphy 3: EF < 50 or history of heart failure and use of digitalis (e.g. Digoxin), non-hydropyridines CCB (Diltiazem, Verapamil).
Arrhythmia Based on Electrocardiogram, (EKG; 1-6) Diagnosed by medical experts	1: No arrhythmia<br>2: Sinus bradycardia (heart rate <60)<br>3: Elongated QTC (> 440 ms in men or > 460 ms in women) on EKG<br>4: Elongated P-R interval >200 m-second on EKG<br>5: Supraventricular, ventricular, or paroxysmal atrial tachyarrhythmia patterns on EKG.<br>6: Atrial fibrillation on EKG
Peripheral Artery Disease (PAD; 1-2)	1: No PAD<br>2: Left or right ankle brachial index (ABI) < 0.9, claudication during walking
**Cerebrovascular Accident (CA)**	
Stroke (1-4)	1: No stroke<br>2: Transient ischemic accident (TIA) with no history of severe stroke<br>3: History of documented stroke, elongated sudden numbness, or loss of speech with no paralysis or limb weakness<br>4: Paralysis or paresis in either limbs or any sudden and long lasting of motor neuron dysfunction such as cranial nerve (CN) CN-III, CN-IV, CN-VI (ocular movements, nystagmus and ocular convergence); CN-IX (Oropharynx symmetry); CN-VII (Facial asymmetry) (diagnosed by neurologist, documented in electronic health record or MyChart).
**Renal (Re)**	
Chronic Kidney Disease (CKD; 1-5) Using CKD-EPI formula	1: Estimated glomerular filtration rate (eGFR) > 90 (no CKD)<br>2: Stage-1: 60 ≤ eGFR ≤ 90<br>3: Stage-2: 45 ≤ eGFR ≤ 59<br>4: Stage-3: 30 ≤ eGFR ≤ 44<br>5: Stage-4: eGFR ≤ 29
**Metabolic (Me)**	
Diabetes Mellitus (DM;1-6)	1: No DM<br>2: Fasting Blood Sugar >110 and less than 126 mg/dL or impaired glucose tolerance test (140 at ≤ 2 hours postprandial blood sugar ≤ 199 (mg/dL)<br>3: History of DM or DM with no complications<br>4: DM with Chronic Kidney Disease (CKD) with no retinopathy<br>5: DM with retinopathy no CKD<br>6: DM with both CKD and retinopathy
Hyperlipidemia (1-3)	1: No hyperlipidemia<br>2: Triglyceride (TG) >150 mg/dL, low density cholesterol (LDL) > 130 mg/dL, high density cholesterol (HDL) < 40 mg/dL for men and < 50 mg/dL for women, and no medication<br>3: TG > 150 mg/dL, LDL > 130 mg/dL, HDL < 40 mg/dL for men and < 50 mg/dL for women, and medication for hyperlipidemia
**Gastrointestinal and Liver (GL)**	

Liver (1-3)	1: No liver disease<br>2: History of hepatitis or increased liver function tests (AST and ALT) 2 times more than maximum normal; AST: Men: 14–20 U/L, Women: 10–36 U/L; ALT: Men: 10–40 U/L, Women: 7–35 U/L<br>3: Liver fibrosis (based on FIB4 scoring system)[4]<br>FIB-4 = Age (years) × AST Level (U/L) / Platelet Count (10^9/L) × √ALT (U/L)>1.30<br>4: Liver cirrhosis
Gastrointestinal disease (GID; 1-3)	1: No GID<br>2: Gastritis or gastroesophageal reflux disorder (GERD)<br>(History of acid reflux, heartburn, indigestion)<br>3: History of peptic ulcer, current ulcer, coffee ground vomit, black stool, or use of antiacid medication
**Respiratory (Res)**	
Chronic Obstructive Pulmonary Disease (chronic bronchitis or emphysema) (COPD; 1-3)	1: No COPD<br>2: Phlegm on most days for 3 consecutive months or more during the year or wheeze with physical exam (1.50≤forced expiratory volume in 1 second (FEV1) ≤1.99; -2.55≤Z-score≤-1.63) in respirometry and no medication use[5,6]<br>3: Phlegm on most days for 3 consecutive months or more during the year or wheeze with physical exam (FEV1≤1.5 L, <50% of predicted value, Z score<-2.55) of predicted value in respirometry) and use of medication (adrenergic drugs, leukotriene receptor antagonists, or others)
Adult-Onset Asthma (1-2)	1: No asthma<br>2: Documented adult onset, still having asthma
**Dysthyroidism (Th)**	
Hypothyroidism[7] (1-4)	1: Euthyroid (Thyroid stimulating hormone (TSH) = 0.3–4.5 mIU/L)<br>2: Subclinical hypothyroidism: 4.5 ≤ TSH ≤ 6.9<br>3: 7.0 ≤ TSH ≤ 9.9<br>4: TSH ≥ 10 or use of levothyroxine
Hyperthyroidism (1-2)	1: Euthyroid<br>2: TSH < 0.3 or use of thiouracil, methimazole or other antithyroid medication
**Hematopoietic System (He)**	
Anemia (1-4)	1: No anemia<br>2: Mild anemia (women: 11.0 ≤ blood Hemoglobin (Hb) level <12 g/dL; men: 11.0 ≤ Hb <13<br>3: Moderate anemia: 8.0 ≤ Hb <11<br>4: Severe anemia Hb < 8.0 (rarely seen in population studies
Thrombocytopenia (1-2)	1: Normal platelet count<br>2: Platelet count < 150,000 without cancer or liver disease states
White Blood Cell count (WBC, 1-3)	1: Normal WBC count<br>2: Leukopenia (WBC<4,000) in blood<br>3: Leukocytosis (WBC>10,000) in blood
**Oral Health (Periodontitis: Pe)**	
Periodontitis (1-3)	1: Normal<br>2: Gingivitis or periodontitis<br>(Increased Pocket depth or attachment loss, bleeding in gum, loose gum)<br>3: Edentulous, >50% tooth loss due to periodontitis
**Musculoskeletal System (MS)**	
Osteoarthritis (OA, 1-3)	1: No OA<br>2: Pain more than 1 month or stiffness but not both, at either knee or hip joints<br>3: Both pain and stiffness at the knee or hip joints
Osteoporosis (based on bone mineral density (BMD) measured by dual-energy X_ray	1: Normal bone mineral density<br>2: Osteopenia (BMD score < -1.0)<br>3: Osteoporosis (BMD score < -2.5) or bisphosphonate medications or Vit D3 medication for osteoporosis

absorptiometry (DEXA) scan, total BMD at either vertebrae L1-4, femoral neck, or trochanter; 1-3)	
Gout (1-3)	1: No gout<br>2: Serum uric acid >7.0 mg/dL or history of gout with no medication<br>3: Serum uric acid >7.0 mg/dL or history of gout with medication (Allopurinol, colchicine)
**Sensory System (Se)**	
Hearing (1- 4)	1: Normal hearing<br>2: Decreased hearing ability from poor to deaf without a hearing aid<br>3: Normal to fair hearing ability with a hearing aid<br>4: Poor hearing with a hearing aid
Eye (1-5)	1: Normal eye function<br>2: Cataract but no glaucoma or macular degeneration in medical history<br>3: Glaucoma but no macular degeneration in medical history<br>4: Macular degeneration but no glaucoma in medical history<br>5: Glaucoma and macular degeneration in medical history
**Central Nervous System (CNS)**	
Depression (1-3) CES-D: Center for Epidemiologic Studies Depression Scale (CES-D)	1: No depression<br>2: CES-D scores ≥ 16 but no use of antidepressants.<br>3: CES-D score ≥ 16 and use of antidepressants
Parkinson's Disease (PD; 1-3)	1: No Parkinson's<br>2: Documented history of PD (Hoehn and Yahr scale), mild symptoms, stage ≤ 3 or no medication<br>3: Stage 4 or 5 PD, moderate to severe symptoms or use of medication
Global Cognitive Impairment (CI; 1-3)	1: Normal global cognition<br>2: Total mini mental state (MMS) < 24<br>3: Documented Alzheimer's disease (combined medical examinations)<br>*Vascular dementia is part of cerebrovascular accident
**Cancer (Can)**	
Lung, Breast, Prostate, Stomach, Pancreatic, Leukemia, Thyroid, Ovary, Squamous Cell Carcinoma, melanoma	1: No cancer<br>2: At least one adult-onset cancer
**Late-life Outcomes**	
Disability	Instrumental activities of daily living (iADL) ≥ 2 or activity of daily living (ADL) >1
Geriatric Syndrome	Experienced two injurious falls in past month, urinary or bowel incontinence
Functional decline SPPB < 9	Short Battery of Physical Performance, which includes chair standing (1–4), walking speed (1-4), and balance (1–4) with total score 12
Mortality	Mortality any time after data used in this analysis

Table S2. System-Specific Health Questions. The organ-specific questions to obtain information on the development of organ-specific diseases and their levels are provided in Table S2 at the end of the supplement.

Table S3. The components of the Health Octo Tool and the Bayesian statistical methods

Components of the Health Octo Tool	Description	Bayesian Statistics
Diseases	For example, the cardiovascular system encompasses various diseases such as hypertension (defined by systolic and diastolic blood pressure above accepted thresholds in geriatric textbooks), ischemic heart disease (clinically diagnosed through ischemic pain and related symptoms on Electrocardiogram [ECG]), peripheral artery disease (assessed via Ankle-Brachial Index), various arrhythmias (identified through ECG), and congestive heart failure (identified by signs and symptoms like shortness of breath, ejection fraction value, and other medical indicators), each with differing levels of severity. Diseases are summarized in Table 1.	-
System	We considered an organ system to exhibit morbidity if it demonstrated at least one of these diseases or deviations from normal health (including subclinical states in certain diseases such as subclinical hypothyroidism).	-
BODN<br>(Body Organ disease Number)	It is crucial to acknowledge that disease diagnosis in medicine relies on a combination of factors, including medical history, physical examination, organ-specific laboratory tests, and treatment protocols. Therefore, the BODN is derived from these diagnoses, which, in turn, are based on a variety of health information sources.	BODN quantifies the number of organ systems with at least one disease. Multiple diseases can contribute to BODN, making it an arithmetic progression with unequal distances between sequential numbers. Consequently, it is always modeled using Bayesian Ordinal Regression.
BSC (Bodily Single-System Clock)	Assesses the contributions of disease severities of single organ system contributing into BODN in terms of posterior coefficient.	A Bayesian Ordinal Regression with a cumulative family was used to assess disease severities contributing to BODN as ordinal values, treating BODN as an ordinal outcome.
BSA<br>(Bodily Single-System Age)	Uses chronological age to regress over BSC proxy of organ-specific rate of aging	Regression of chronological age over BSCs was performed using a Gamma distribution with a log link function and a weak prior in the Bayesian framework, yielding the predicted value for each organ as its BSA.

Body Clock	Measures health entropy collectively using diseases and their levels in the model and longitudinal BODN as an outcome. Body Clock is a Bayesian weighted multimorbidity metric in each individual.	A Multilevel Ordinal Regression analysis was used to capture the heterogeneous effects of diseases severities on BODN over time, acknowledging that BODN is a progressive value with unequal distances between consecutive values. The predicted value obtained from the model was termed Body Clock.
Body Age	Rate of aging in the whole bodily organ system health entropy.	Gamma distribution with a *log link* function was employed to regress chronological age over Body Clock, and the predicted value was termed Body Age.
Speed-Body Clock	The effect of Body Clock on Walking Speed as an age-related functional phenotype, considering effects of bodily system health entropy on this functional outcome.	A Gaussian distribution was used to regress walking speed over Body Clock, including the residual variance of walking speed over Body Clock. The predicted values were termed Speed-Body Clock.
Speed-Body Age	Chronological age conditioned on the Speed-Body Clock, as a proxy of related rate of aging.	A Gamma distribution with a *log link* function was used to regress chronological age over Speed-Body Clock, yielding the predicted values termed Speed-Body Age.
Disability Index (DI)	Includes the number of functional and cognitive deficits, urinary and bowel incontinence, and fall as number of events conditioned over the total numbers of items measured (Table S2).	Zero Inflated Beta Binomial
Disability-Body Clock	The effect of Body Clock on DI, indicating the effect of bodily organ system health entropy on this disability metric.	A Negative Binomial model in the Bayesian framework was used to predict DI using the Body Clock, with the predicted value termed Disability-Body Clock.
Disability-Body Age	Chronological age conditioned on the effect of bodily organ system health entropy on disability, indicating related rate of aging.	A Gamma distribution with a *log link* function in the Bayesian framework was used to regress chronological age over Disability-Body Clock. The predicted values were termed Disability-Body Age.

Table S4. Disability Items used in BLSA (47 items). Each impairment is coded as 1.

Slowness
Feeling lonely
Feeling happy (recoded)
I could not get going
Everything was an effort
Difficulty for transportation
Difficulty in financial management
Difficulty using telephone
Difficulty shopping
Difficulty taking medications
Difficulty preparing meal
Difficulty bathing
Difficulty bathing
Difficulty using toilet
Difficulty doing heavy housework
Difficulty doing light housework
Difficulty eating
Difficulty dressing
Difficulty walking
Difficulty doing laundry
Injurious Fall
Urinary incontinence
Owel incontinence
Orientation on today's date
Orientation on Month
Orientation on year
Orientation on which day of week is today
Orientation on Season
Orientation on Place (name of place)
What floor
What city are we in?
What state are we in?
What country are we in?
Immediate recall on object one (ball)
Immediate recall on object two (flag)
Immediate recall on object three (tree)
Count backward from 100 by 7 (each time is scored, stop after 5 subtractions) or spell the WORLD backward
Recall three previous objects (each object recall is one item).
Nine items for language
Show wristwatch to the participant and ask what it is.
Show pencil to the participant and ask what it is
Ask to repeat "No", "If" "and", and "but"
Give the participant a piece of paper and say "Take this paper in your right hand and fold it in half and put it on the floor.
Ask the participant to read "Close your eyes" and to do what it says.
Give a piece of paper to the participant and ask to write a sentence spontaneously.
Ask the participant to copy an intersecting pentagon.

Statistical Analyses

Bayesian inference. We used a Bayesian approach in developing our new multimorbidity tool. A Bayesian model comprises two components: 1) Prior knowledge on the estimates (parameters), as the information before observing the data $P(\Theta)$ where Θ denotes the parameters; and 2) the likelihood $P(Y|\Theta)$ of the information contained in the data (Y). Using the Bayes formula, we can obtain the posterior distribution of the parameters $P(\Theta|Y)$, which can be updated when encountering new data. Moreover, the Bayes approach provides a distribution of estimates rather than a point estimate, and uncertainty around the estimates is known as the credible interval (CI)[8,9].

We used BODN as an ordinal outcome and time-1 or time-2 disease levels as lagged ordinal predictors, adjusting for time but excluding chronological age (c-age). We used multilevel ordinal regression and individuals as model levels in a Bayesian framework[9] to develop various models, assessed the models' predictive accuracy using leave-one-out cross-validation (LOO-CV)[10], quantified model weights, and compared all models with c-age and time. Including all disease levels in one model, we obtained post-analyses predicted BODN at individual levels to quantify individual Body Clocks.

We evaluated the models using in-sample (using the BLSA parameters to predict BODN in simulated data) and out-of-sample accuracy (using BLSA parameters to predict BODN in InCHIANTI data) with "predictive checks," as part of the usual Bayesian workflow [11]. We then determined the model-specific weights, performed model comparisons using the Bayesian stacking approach, and compared all models' weights with the age-only model. We used the Bayesian Negative Binomial distribution to predict late-life outcomes.

Bayesian Approach for Multilevel Ordinal Outcome and Ordinal Predictors. With ordinal outcomes, the difference between the orders might not be equidistance. In other words, moving from having one organ with at least one disease to the next organ with disease is not necessarily equal to moving from having two or three organ morbidities to the next organ morbidity. We used a Bayesian ordinal model that assumes the observed ordinal outcome Y (i.e., BODN) is derived from categorizing a latent continuous variable $\tilde{Y}$, here $\widetilde{\text{BODN}}$ [12]. Therefore, we used longitudinal, multilevel ordinal regression models with BODN as an ordinal outcome and each person as a level using the brms Bayesian software package, built on top of STAN, a probabilistic programming framework for Bayesian inference[13-15]. Also, considering that the levels of ordinal predictors might not be equidistant, we used a function called "monotonic effect" implemented in the Bayesian brms software package published previously, which assumes that the ordinal predictor is a latent continuous variable with a posterior estimate (total beta coefficient for the ordinal variable). When encountering information from the data, a cut-point is introduced using the "simplex function" ζ, so that $\zeta\, i \in [0,1]$. Therefore, ζ is the posterior probability of each level, and *i* is the number of ordinal categories. We can quantify the posterior estimate (beta coefficient) of each disease level by multiplying the total posterior estimate by the proportion of each level ζi, e.g., HTN has three levels, the first level is the reference [normal]; the second and the third level proportions are ζ% and 1-ζ%, respectively. With the posterior estimate ϴ (total beta coefficient) of the latent continuous HTN, the posterior estimate of each HTN level would be (ϴ * ζ%) and (ϴ * 1- ζ%), respectively[13]. Except for peripheral artery disease (PAD), hyperthyroidism, cancer, thrombocytopenia, and asthma, which are binary, all other diseases are ordinal (Table S2).

Using all disease-level burdens, we obtained predicted BODN for each individual as a level, termed Body Clock, at each time-point and change in Body Clock over time. We used change in Body Clock to predict late-life outcomes such as Short Battery of Physical Performance (SBPP), geriatric syndrome, disability and mortality, using multilevel Negative Binomial Models in a Bayesian inference framework. The models are summarized in Table S3.

Prior Knowledge of Predictive Estimates of BODN. We determined the prior distribution for each parameter, including intercept and beta coefficients using weak priors. For the beta coefficients and intercepts classes, the prior estimate with a normal distribution (mean: $\mu = 0$, variance $\sigma = 10$) was used, and for the class standard deviation (sd) related to varying subjects, the half-Cauchy (0,10) was used. The uniform prior with a Dirichlet distribution was used for ordinal predictors (i.e., (2, 2) for $\zeta 1$ and $\zeta 2$ [simplex parameters] for a three-level predictor)[13]. We used a dynamic Hamiltonian Markov chain Monte Carlo (MCMC) algorithm[9,15] to obtain posterior draws. We performed the analyses with a minimum of five chains and a minimum of 5,000 iterations. We obtained mean posterior values from the posterior distribution of the parameter estimate Θ if the distributions were normal but reported the median if not normal.

Markov Chain Monte Carlo (MCMC). MCMC is a class of algorithms used in Bayesian statistics to approximate the posterior distribution of parameters when direct calculation is difficult or impossible. In Bayesian inference, we are interested in the posterior distribution $p(\theta|y)$, which combines the prior $p(\theta)$, and the likelihood $p(y|\theta)$ described above. However, for complex models, computing this distribution analytically is often infeasible. MCMC solves this by generating a sequence of samples from the posterior distribution using a Markov chain—a process where the next sample depends only on the current one. After enough iterations, the distribution of the sampled values closely approximates the posterior, allowing us to estimate

parameters and quantify uncertainty even in complex Bayesian models[9]. There are different types of MCMC. In STAN and brms, a Hamiltonian algorithm is implemented [15].

Model Inference Diagnostics. To assess the computational success and resulting inferences, we evaluated the convergence diagnostic Rhat and the effective sample size measures for each estimated parameter[16].

Model Comparisons. Each Bayesian model has its own predictive performance on a logarithmic scale called the estimated log predictive density (ELPD). The ELPD estimates the predictive performance of future data, and its standard error (SE) quantifies the uncertainty in knowing the future BODN exactly. Leave-one-out cross-validation (LOO-CV)[10], a variant where each observation takes the role of the validation set in turn and leads to a natural single prediction approach, was used for each model evaluation and also for model comparisons. Pareto smoothed importance sampling (PSIS) was used for faster LOO-CV estimates[10]. PSIS is based on the concept that problematic parts of the posterior distribution can be identified by evaluating the importance sampling weights. It checks for tails of the distribution where importance sampling may be particularly unreliable. If the PSIS diagnostic indicated an unreliable PSIS estimate ($k>0.7$), we reassess the model and MCMC for a slower but more accurate computation. To compare each model with the age-only models, we used LOO-CV and the models' ELPD comparisons to obtain ELPD_DIFF, which shows ELPD differences between each model and their paired age-only models. The models with larger ELPD are optimal, and a negative ELPD_DIFF favors the first model in the comparison.

Model Evaluation and In-Sample and Out-of-Sample Predictive Checks. To assess the robustness of the model to predict BODN, we used the time-1 full model based on BLSA data to predict BODN in the InCHIANTI and NHANES data as out-of-sample validations. We used

"posterior predictive checking"[11] implemented in the brms software package to compare the posterior predictive density of simulated data to density estimates of the observed data (in-sample evaluation of BLSA) or predict new data (InCHIANTI data; out-of-sample validation).

Bayesian Stacking Weights to Compare Models. Bayesian stacking is useful for computing model weights for several models simultaneously by optimizing the estimated LOO-CV predictive performance of the weighted model combinations. Here we computed stacking weights for each kind of model paired with their corresponding age-only models (single-disease, single-system, stepwise multisystem, and full models separately). The Bayesian stacking approach provides model-specific weights versus the paired age-only models and identifies the models with the best predictive performance.

Zero Inflated Beta Binomial. We utilized the Zero-Inflated Beta Binomial model to create the new Disability Index, Disability-Body Clock, and Disability-Body Age. Initially, a Bayesian-based beta binomial model was developed to calculate the probability of events (in this case, late-onset outcomes) given the number of trials (which represented the total number of disability items, 47 items in this context) using the formula (S1) below:

(S1) $P(\text{event}|\text{trials}, p) = \binom{\text{trials}}{\text{event}}\ p^{\text{event}}\,(1-p)^{\text{Trials}-\text{event}}$

$$p_{\text{i}=} \frac{\exp(\eta_i)}{1+\exp(\eta_i)}$$

p is probability of the event for observation i and η_i is the linear predictor term.

For mixing the zero inflated distribution to the beta binomial the formula (S2) is:

$$(S2)\ p(\text{event}|\text{trails}, \mu\Phi, (1-\mu)\Phi, \theta)$$

$$= \begin{cases} \theta(1-\theta)\text{Beta Binomial (event} = 0|\text{trials}, \mu\Phi, (1-\mu)\Phi) \\ (1-\theta)\ \text{Beta Binomial (evet} > 0|\text{trials}, \mu\Phi, (1-\mu)\Phi) \end{cases}$$

μ: standard Beta Binomial distribution
Φ: densities of values

Θ: probability of zero values for the events
$(1-\theta)$: probability of the event values more than zero

Body Clock and late-onset outcomes

To predict disability and other late-onset outcomes and mortality across all age groups using the Negative Binomial statistical family that accounts for many zero values, we used the Body Clock to predict these binary outcomes. The Body Clock enables the differentiation of resilient and non-resilient individuals in developing such late-onset health outcomes.

Supplemental Results.
Table S5 depicts the descriptive baseleine characteristics of BSLA by baseline age groups.

Table S5. Baseline Characteristics of BSLA participants

	**20-44 (n=68)**	**45-55 (n=137)**	**56-64 (n=230)**	**65-74 (n=278)**	**75-85 (n=143)**	**>85 (n=51)**
Follow-up (years) Mean ± SD (range)	5.8±4.1 (1-15)	5.4±3.7 (1-15)	4.1±2.7 (1-14)	3.5±2.2 (1-14)	5.0±0.37 (1-9)	5.2±0.7 (1-11)
Sex						
Women %	51.5	39.4	38.7	56.8	62.2	51.0
**Cardiovascular System (CV)**	19.1	27.0	44.8	54.3	59.5	62.7
HTN	0	2.9	10.4	14.4	28.7	25.5
IHD	36.8	40.2	50.4	51.8	60.2	80.4
CHF	27.9	24.8	33.0	42.8	74.5	49.0

Arrhythmia<br>PAD	2.9	1.46	0.87	2.9	9.1	13.7
**Cerebrovascular Accidence (CA)**	4.4	4.4	4.3	9.4	17.5	17.6
**Renal (Re)**	32.4	66.4	73.1	92.8	100	96.1
**Metabolic System (Met)**<br>DM<br>Hyperlipidemia	17.6<br>35.3	26.3<br>28.5	39.1<br>33.5	43.2<br>28.0	48.3<br>21.85	53.1<br>29.4
**GI-Liver (GL)**<br>Liver<br>GID	2.9<br>4.4	11.7<br>16.8	13.9<br>20.0	16.6<br>21.6	32.9<br>25.2	15.7<br>15.7
**Respiratory System (Res)**<br>COPD<br>Adult Asthma	0<br>8.8	2.2<br>11.0	1.6<br>6.5	4.7<br>4.7	9.1<br>7.0	11.8<br>3.9
**Dysthyroidism (Th)**<br>Hypothyroidism<br>Hyperthyroidism	5.8<br>0	12.4<br>2.9	14.8<br>0	15.5<br>0.7	24.5<br>2.1	25.5<br>3.9
**Hematopoietic Diseases (He)**<br>Anemia<br>Thrombocytopenia	10.3<br>5.6	8.0<br>2.9	6.0<br>3.5	8.6<br>5.0	15.4<br>6.3	23.0<br>3.9
**Periodontitis (Pe)**	20.6	22.6	34.8	35.3	34.3	23.5

**Musculoskeletal System (MS)**	23.5	40.1	50.0	45.3	44.0	37.3
Osteoarthritis	23.5	31.4	41.3	54.0	66.4	90.0
Osteoporosis						
**Sensory (Se)**						
Hearing	7.4	9.5	13.5	27.0	53.9	54.9
Eye	4.4	9.5	25.3	61.5	82.5	94.1
**Central Nervous System (CNS)**						
Depression	25.0	16.1	8.3	9.4	14.7	13.7
Parkinson's	0	0	0.87	0.4	2.1	0
Cognitive Impairment	0	0	0	0.4	0.7	17.7
**Cancer (Can)**	5.9	5.1	7.8	17.6	18.2	16.7
**Events Over the Course of Study**						
SBPP < 9.0	0	1.2	1.9	5.6	20.2	66.7
Disability	0	1.5	1.3	4.7	18.3	66.7
Geriatric syndrome	0	0	1.1	34.58	61.02	86.32
Mortality	0	0.49	0.43	1.4	3.3	5.9

SD: standard deviation, CVS: cardiovascular system, HTN: hypertension, IHD: ischemic heart disease, CHF: congestive heart failure, PAD: peripheral artery disease, CVA: cerebrovascular accidents, CKD: chronic kidney disease, DM: diabetes mellitus, GID: gastrointestinal disease, COPD: chronic obstructive pulmonary disease, MS: musculoskeletal diseases, CNS: central nervous system, SBPP: a short battery of physical performance.

Body Organ Disease Number (BODN)

BODN considers 11 organs and two disease states, cerebrovascular incidents and cancer. The predicted BODN at an individual level is quantified and is termed the Body Clock. Clockwise, the Body Clock includes the central nervous system and cerebrovascular accidents, cardiovascular system, respiratory system, gastrointestinal and liver system, metabolic system, cancer, renal system, hematopoietic system, the sensory systems (ophthalmic and hearing), dysthyroidism, and the musculoskeletal system (Fig S1.)

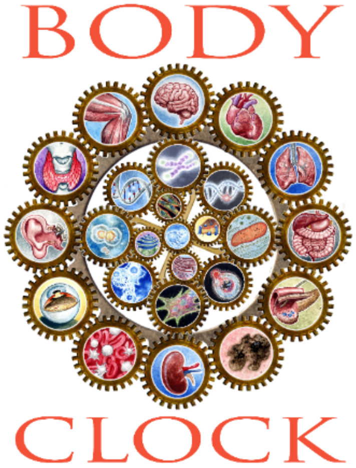

Fig S1. Symbolic representation of the integrated system-based calculated Body Organ Disease Number[17] and suggestive plausible cellular physiology underlying the BODN phenotype.

The Distribution of Organ Systems in the Body Organ Disease Number (BODN) over a Decade Shows Heterogeneity.

The interactive sunburst graphs illustrate that the cardiovascular system (CV) exhibited the highest frequency as components of the BODN across all age groups (Fig. S2). As individuals age, other organ systems, such as the kidney, sensory system, and musculoskeletal system, show an increasing presence in BODN. Those with cancer tend to exhibit higher levels of multisystem morbidity even in younger individuals. Overall, the patterns of multisystem frequencies in all age

groups appear to be random, however, multisystem frequency does not delineate the magnitude of their effect on the body.

Body Organ Disease Patterns

The interacting sunburst graphs for the InCHIANTI are illustrated below and can be accessed via the link given. In the youngest age group (≤ 44 years), >50% of individuals had no morbidity or morbidity in one organ system only. In this age group, participants with cancer frequently have other system morbidities, including cardiovascular system (CV), central nervous system (CNS), and renal system (Re) (Fig. S2a). With increasing age, BODN increases in heterogeneous combinations so that both the number of people with multiple organ-system morbidities and the number of systems with morbidity increase. Particularly, periodontitis (Pe), metabolic (Me), renal (Re), respiratory (Res), and dysthyroidism (Th) become more frequent after age 55; CV maintained its increased frequency (Fig. S2c). Re, sensory (Se), and musculoskeletal (MS) systems appeared more frequently after age 65 (Fig. S2d), while other morbidities such as cerebrovascular incident (CA), cancer (Can), gastrointestinal and liver (GL), and Res also increased. After age 75, most persons showed multiple organ system morbidities, with sensory (Se), CNS, and MS appearing more frequently. CV, however, was still the most frequent system morbidity in all age groups.

Fig. S2. Frequency and Combination Patterns for Body Organ Disease Number in InCHIANTI. The interactive graphs illustrate any existing combined multisystem morbidities. **For more detailed information and interactive graph please click on the link.**

[Body Organ Disease Patterns (shinyapps.io)]

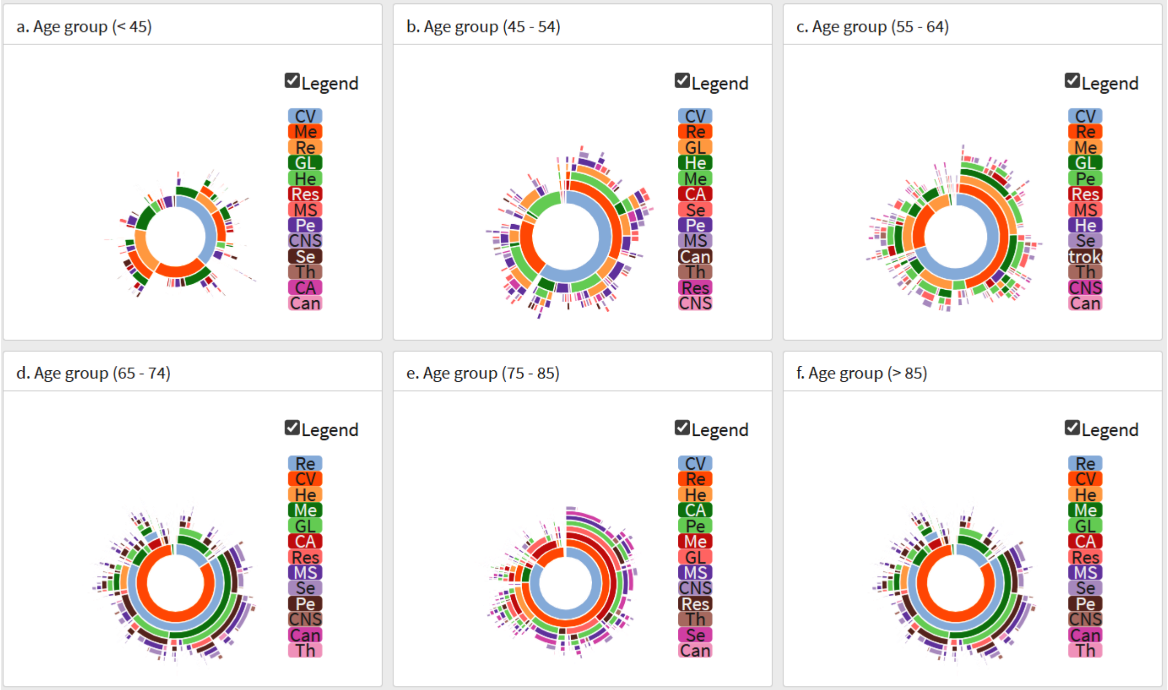

Bodily System-Specific Age

In both BLSA and InCHIANTI datasets, the correlations of BSA values are diverse, and organ systems do not age uniformly or simultaneously (Fig. S3A & B). The comparison between BSA and chronological age reveals that biological aging for some systems can exceed chronological age (Fig.S3C). In the InCHIANTI population, data are concentrated around 65-85 years old, creating a gap in the graph from the younger age range. The posterior estimates and their uncertainty measures (95% credible interval) of maximum latent corporation of each disease and their levels into longitudinal BODN in single disease, single system, and system entropy including all systems in both BLSA and InCHIANTI data are presented in Table S6 A-I. The

model weights for single disease, single system and entropy model (all systems) reported in Table S7 A-D.

Fig S3. Bodily System-Specific Age (BSA) represents rate of aging of each organ systems. BSA was developed regressing chronological age over Bodily System Clocks (BSCs). A BLSA; B InCHIANTI; C. BSA vs. chronological age.

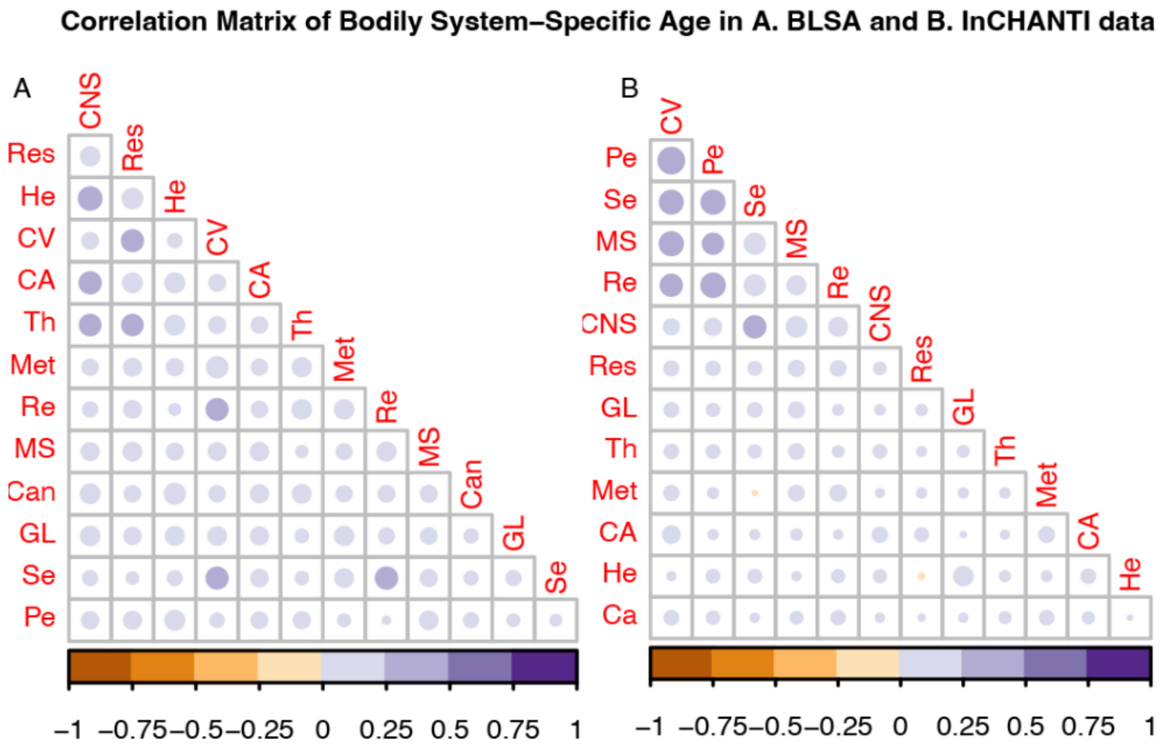

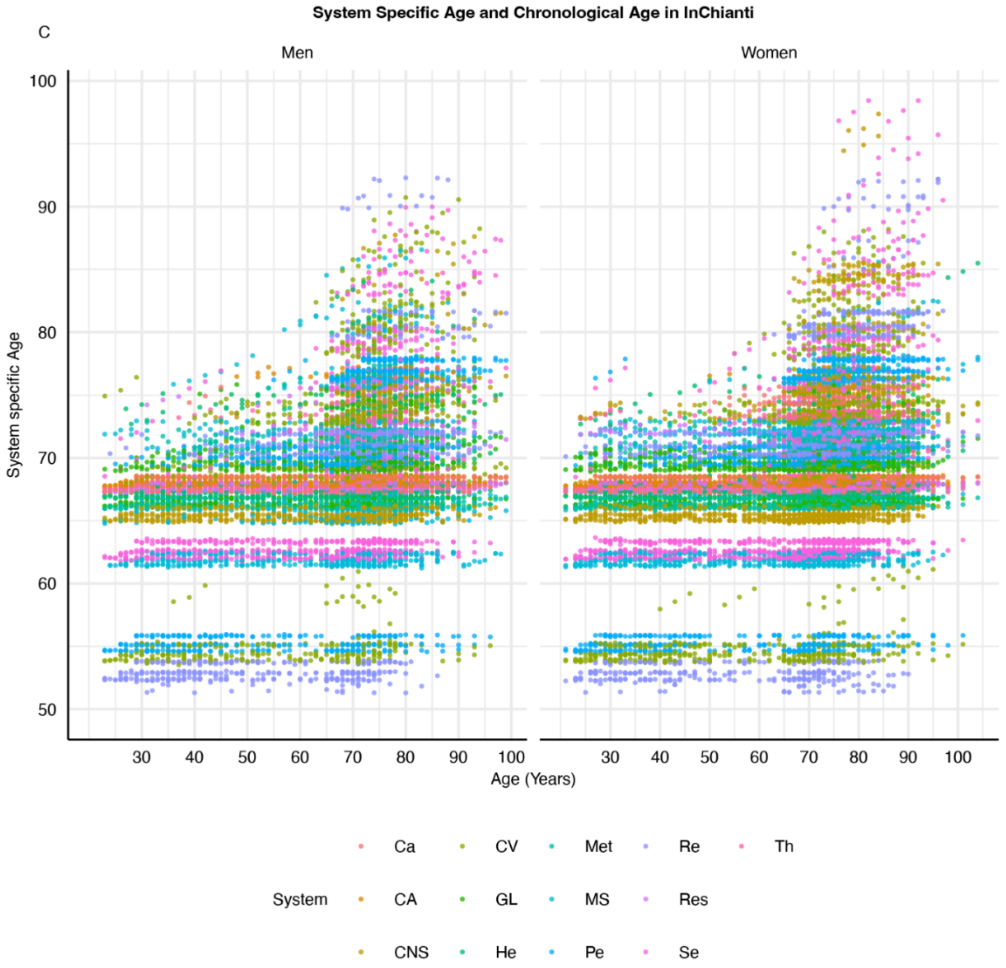

Heterogeneous incorporation of disease levels into longitudinal BODN

The effect of disease levels or severity incorporating into longitudinal BODN was obtained. With all disease levels in the model, the posterior estimates of each disease in the same organ become smaller due to shared pathophysiology. The milder states of organ-system diseases had larger estimates contributing to longitudinal BODN compared to more severe states of these diseases (Fig.S4A&B, Table S6). Similarly, diseases without pharmacological treatment had larger posterior coefficient estimates for predicting BODN (Fig. S4A&B Table S6). For example, HTN without treatment was a stronger contributor to BODN (b=0.32, 95% CI: 0.23–0.39) than HTN with treatment (b=0.17, 95% CI:0.13–0.22). Similarly, congestive heart failure with preserved

ejection fraction had a larger posterior coefficient estimate (b=0.22, 95% CI:0.12–0.23) compared to the congestive heart failure with a low ejection fraction (b=0.09, 95% CI: 0.05–0.13). Arrhythmias, such as sinus bradycardia, elongated QTc, and atrial fibrillation, also significantly affected BODN (Fig. S4A&B; Table S6C, F&I). Stage-1 age-related chronic kidney disease (CKD), decoupled from diabetic kidney failure, exhibited stronger incorporation into BODN (b=0.53, 95% CI: 0.38–0.71) compared to stage-2 (b=0.07, 95% CI:0.05–0.10) and stage-3 CKD (b=0.1,95% CI:0.05–0.10), as well as end-stage renal disease (b=0.20, 95% CI:0.15-0.28).

Fig. S4 The disease-level coefficient estimates that are heterogeneously incorporated into BODN in the full models. Significance was determined when the 95% CI does not include 0. A Time 1 maximum posterior estimates of disease levels incorporating into longitudinal BODN. B Time 2 maximum posterior estimate of disease levels incorporating into longitudinal BODN. Abbreviations: HTN: hypertension, IHD: ischemic heart disease, CHF: congestive heart failure, Arr: arrhythmia, PAD: peripheral artery disease, CKD: chronic kidney disease, DM: diabetes mellitus, GID: gastrointestinal disease, COPD: chronic obstructive pulmonary disease, Hypoth: hypothyroidism, Hyperth: hyperthyroidism, OA: osteoarthritis, Osteop: osteoporosis, Periodon: periodontal disease, Dep: depression, Park: Parkinson's disease, CI: cognitive impairment. Systems abbreviations: CV: cardiovascular system, CA: cerebrovascular accidents, Re: renal system, Me: metabolic, GL: gastrointestinal and liver, Res: respiratory, Th: Thyroid, He: hematopoietic, MS: musculoskeletal, Pe: periodontitis, Se: sensory, CNS: central nervous system, Can: cancer.

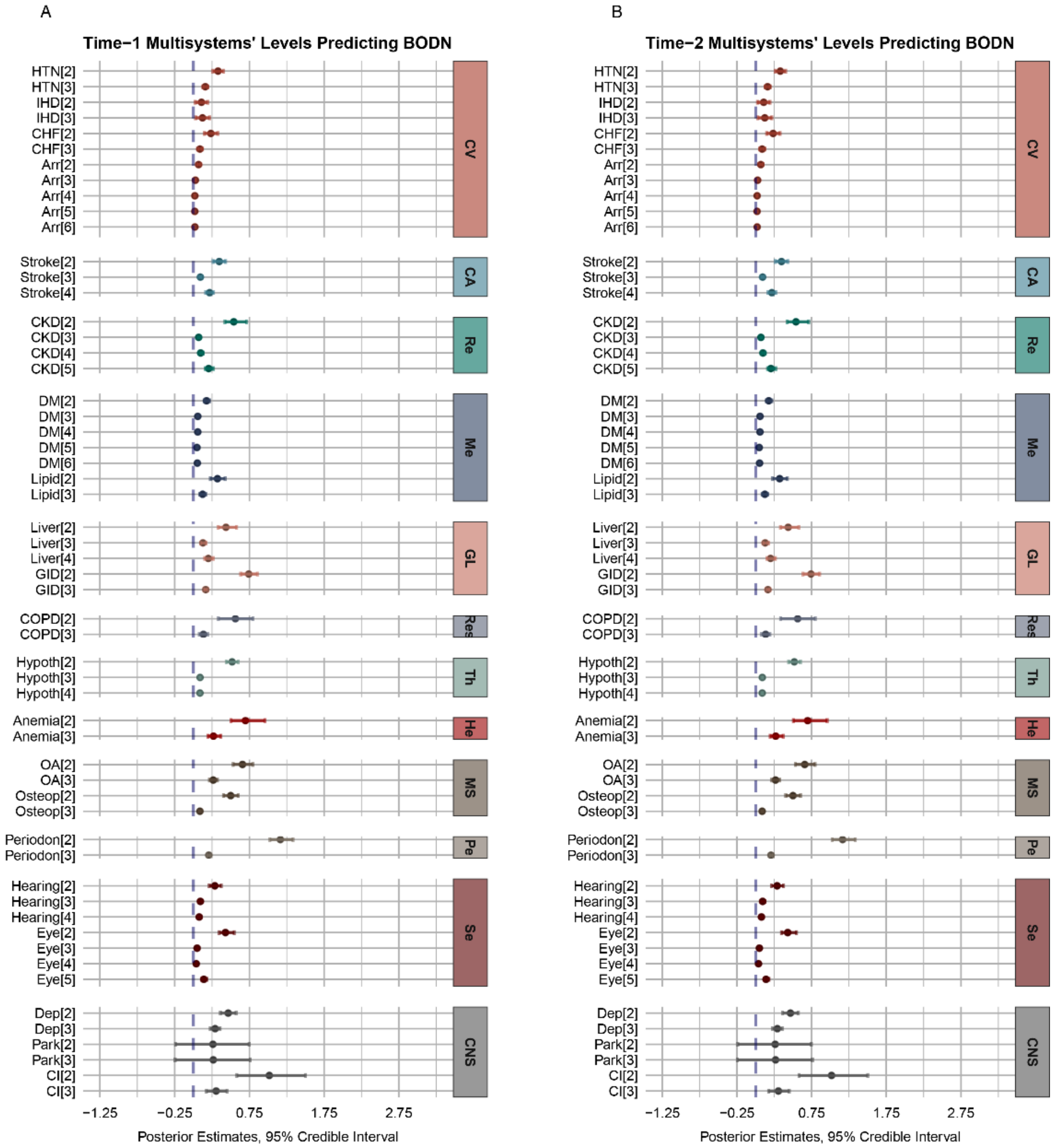

A
Time−1 Multisystems' Levels Predicting BODN
B
Time−2 Multisystems' Levels Predicting BODN
HTN[2]
HTN[3]
IHD[2]
IHD[3]
CHF[2]
CHF[3]
Arr[2]
Arr[3]
Arr[4]
Arr[5]
Arr[6]
CV
Stroke[2]
Stroke[3]
Stroke[4]
CA
CKD[2]
CKD[3]
CKD[4]
CKD[5]
Re
DM[2]
DM[3]
DM[4]
DM[5]
DM[6]
Lipid[2]
Lipid[3]
Me
Liver[2]
Liver[3]
Liver[4]
GID[2]
GID[3]
GL
COPD[2]
COPD[3]
Res
Hypoth[2]
Hypoth[3]
Hypoth[4]
Th
Anemia[2]
Anemia[3]
He
OA[2]
OA[3]
Osteop[2]
Osteop[3]
MS
Periodon[2]
Periodon[3]
Pe
Hearing[2]
Hearing[3]
Hearing[4]
Eye[2]
Eye[3]
Eye[4]
Eye[5]
Se
Dep[2]
Dep[3]
Park[2]
Park[3]
CI[2]
CI[3]
CNS
−1.25
−0.25
0.75
1.75
2.75
Posterior Estimates, 95% Credible Interval

Leave-one-out cross-validation to assess model performance

Leave-one-out cross-validation (LOO-CV) is a method where each observation is sequentially treated as the validation set, allowing for a natural single-prediction evaluation. This approach was applied for both model assessment and comparison. To accelerate LOO-CV calculations, Pareto smoothed importance sampling (PSIS) was employed. PSIS helps identify problematic regions in the posterior distribution by analyzing importance sampling weights, particularly in the distribution tails where sampling may be less reliable. The acceptable PSIS diagnostic estimate (k <0.7) is reliable (Fig. S5).

Fig S5. Leave-one-out cross-validation (LOO-CV) for model assessment (with diagnostic k < 0.5). Full models leave-one-out cross validation in at A time-1 and B time-2 using BLSA data and C InCHIANTI data. Each + represents an individual at each time-point, (907×3=2701 observations at time-1 and 907×2=1904 at time-2). The Pareto-smoothed importance sampling diagnostic k <0.7 indicates that the LOO-CV computation is reliable and there are no outlier observations.

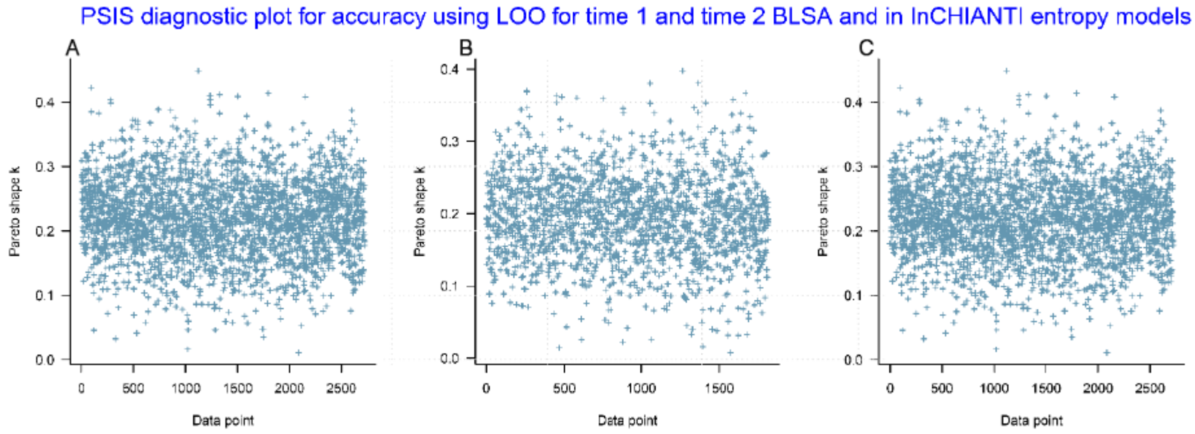

Body Clock Trajectories with Age

We developed the individualized Body Clock by extracting the predicted value of BODN from the post hoc analysis of multilevel ordinal regression model that included all organ systems diseases and their severities for each individual. The individualized trajectory plots for the Body Clock vividly demonstrate diverse trajectories among individuals of the same chronological age. These trajectories exhibit varying slopes and magnitudes, underscoring the unique maximum intrinsic aging experienced by different individuals (Fig. S6). Each individualized trajectory is from maximum monotonic effect of diseases incorporated into BODN.

Fig. S6 Trajectories of the individualized Body Clock with chronological age. A BLSA. B. InCHIANTI data.

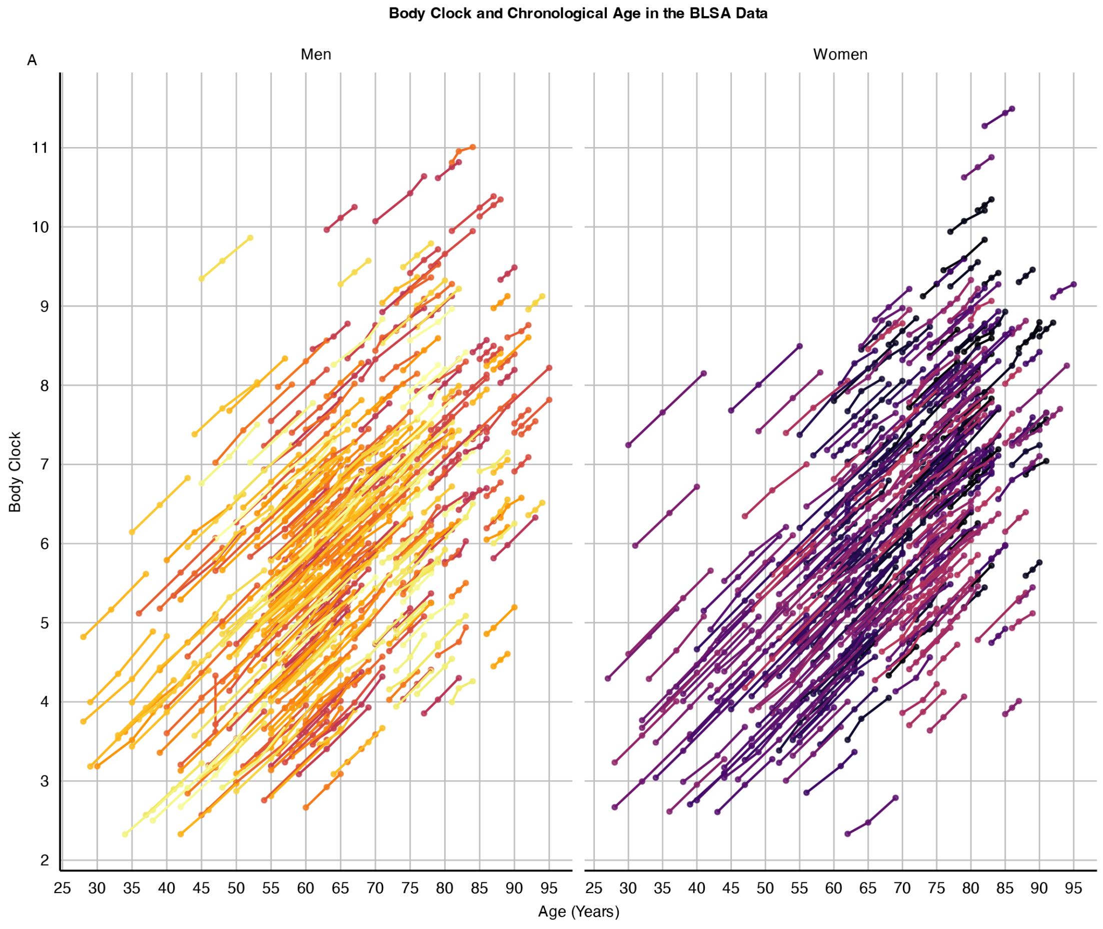

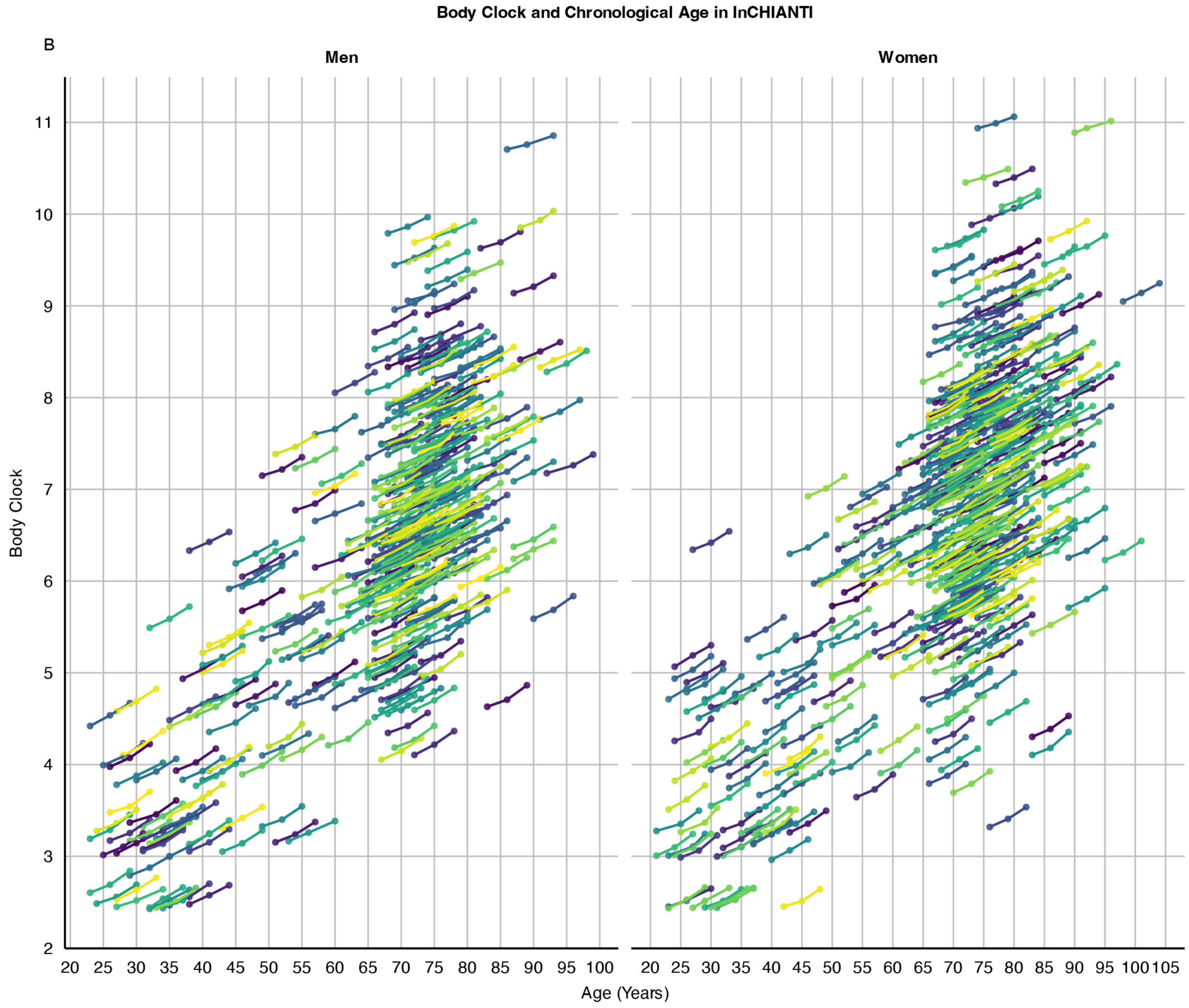

Body Age and Chronological Age

Body Clock increases with each successive 10-year age interval, and its values are heterogeneously distributed within each age group. With increasing age, the probability of larger Body Clock values increases (Fig. S7 A& B). We regressed chronological age over the Body Clock and obtained the predicted value as Body Age using a Gamma distribution family and a log link function (Fig. S7C&D).

Fig. S7 The Body Clock increases with each successive 10-year age interval, and its values are heterogeneously distributed within each age group. A BLSA: <45 years old: Reference group, 45–55 years old: mean=1.40 (95% CI:0.16 –1.09); 56–65 years old: mean= 2.76, 95% CI (2.46–3.05); 66-75 years old: mean=4.56, 95% CI (4.25–4.88);76–85 years old: mean= 6.16 , 95% CI(5.81–6.51); >85 years old: mean= 8.22, 95% CI (7.82–8.62). B InCHIANTI: <45 years old: Reference group, 45-55 years old: mean=4.07 (95% CI:3.75–4.39); 56-65 years old: mean= 4.84, 95% CI (4.5–5.18); 66–75 years old: mean=5.5, 95% CI (5.15–5.86);76–85 years old: mean= 7.9 , 95% CI(7.49–8.31); >85 years old: mean= 10.43, 95% CI (9.97–10.89)

C-D Body Age increases with chronological age. There is inter-individual heterogeneity in the Body Age. C Body Age trajectories in the BLSA. D Body Age trajectories in the InCHIANTI.

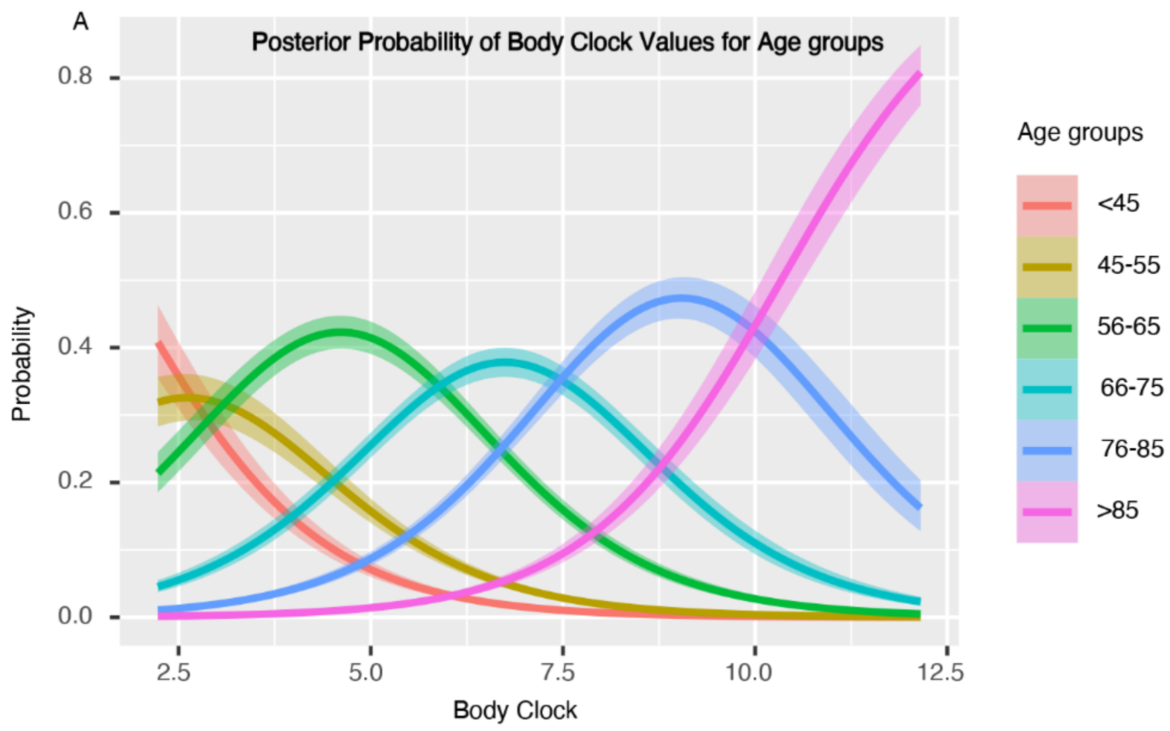

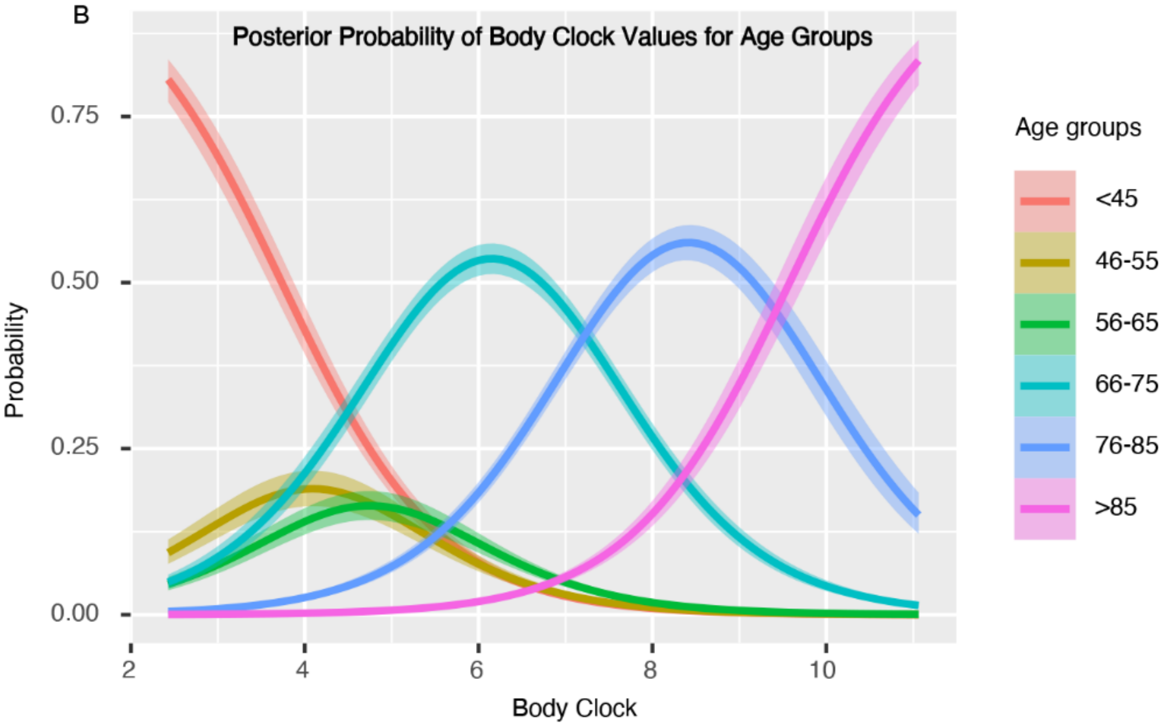

Body Age and Chronological Age in BLSA Data

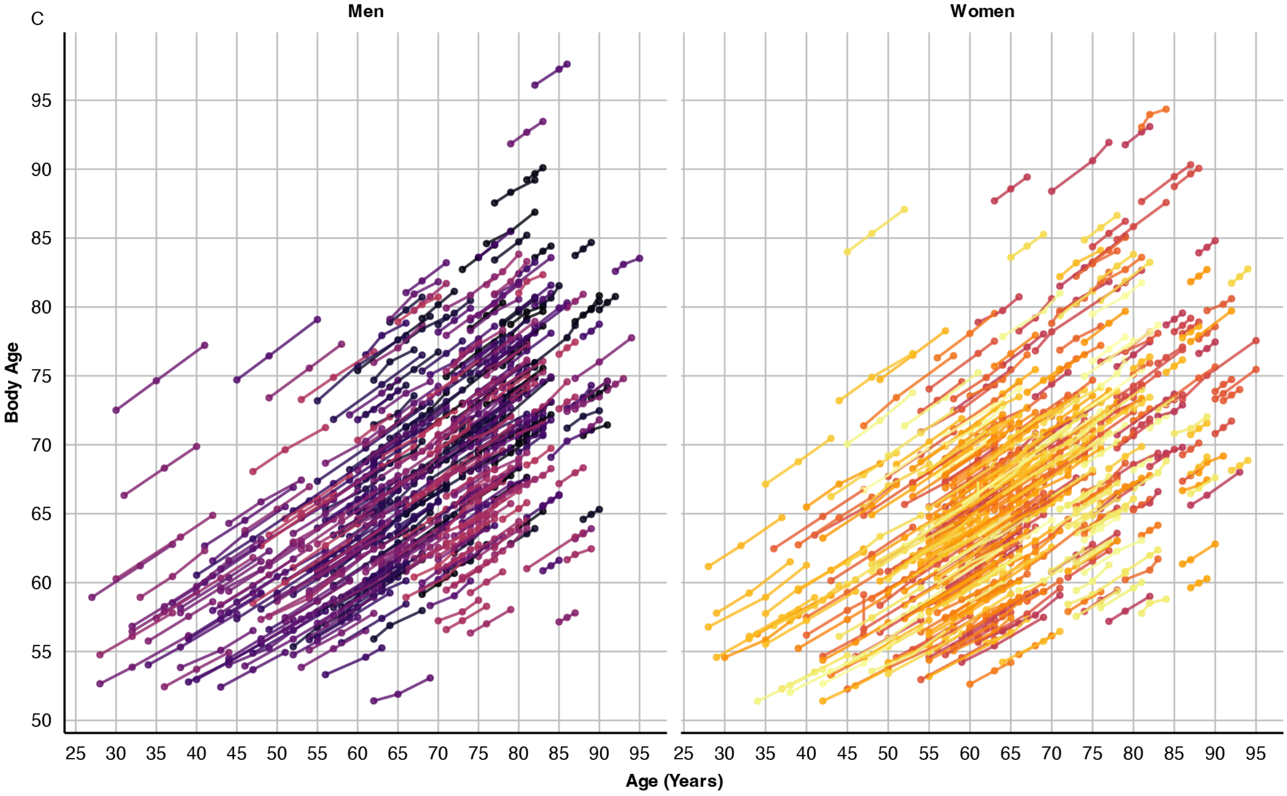

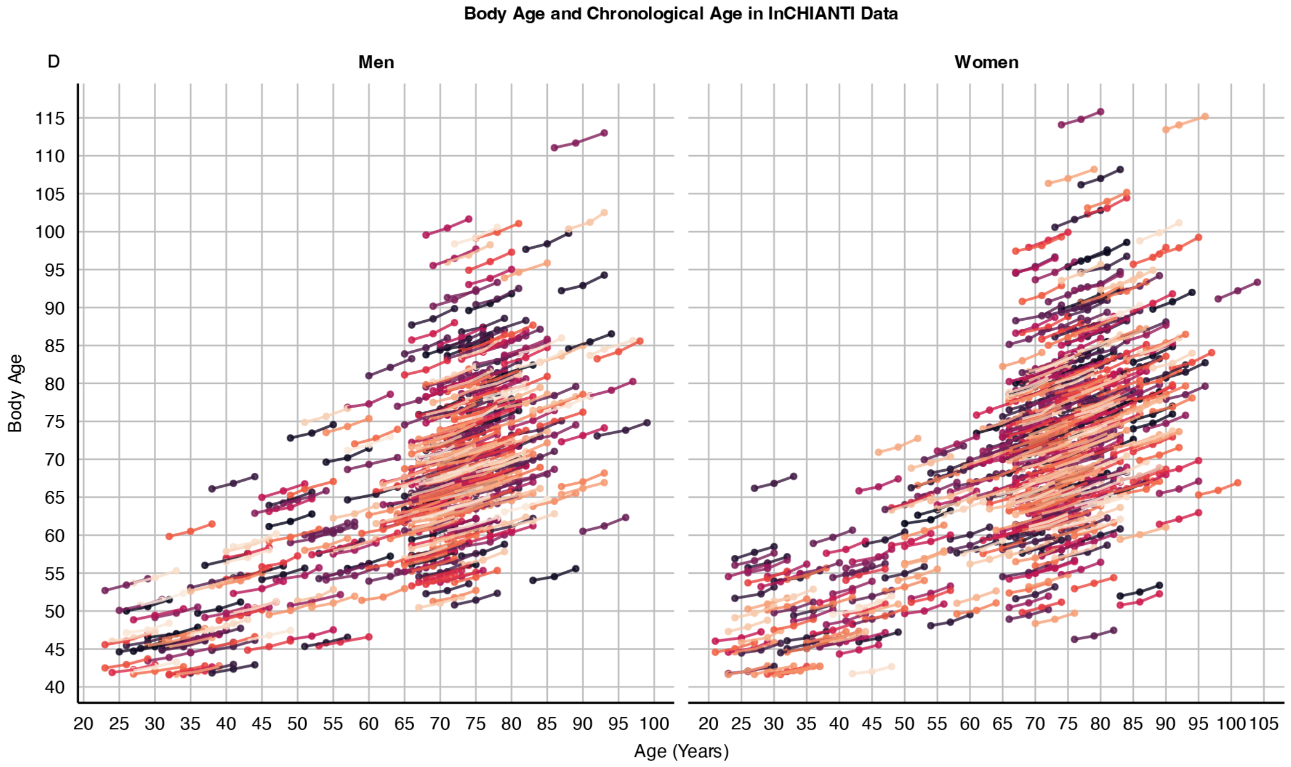

Speed-Body Clock and Speed-Body Age

Speed-Body Clock, obtained from predicting walking speed using the Body Clock, demonstrates an inverse association with chronological age. The Body Clock negatively associated with physical function measured using walking speed. In the BLSA study, women had a lower Speed-Body Clock than men, even after height adjustment **(**1.09±0.1 vs. 1.15±0.1). Each unit increase in Body Clock was associated with a walking speed decrease of -0.062 m/s (men) and -0.064 m/s (women)**.** Similarly, in the InCHIANTI study, a one-unit increase in Body Clock corresponded to a -0.03 m/s reduction in walking speed in both sexes. Women again showed a lower Speed-Body Clock than men (1.29 ± 0.15 vs. 1.56 ± 0.14) (Fig. S8A, S8B). Speed-Body Age was developed regressing chronological age over Speed Body Clock. With a one unit increase in Speed-Body Clock the Speed-Body Age decreases in both BLSA (intercept=5.34, b=-0.99, 95% CI (-1.06 – -

0.95) and InCHIANTI (intercept=4.8, b=-0.43, 95% CI= -0.48 – -0.39). That is, using a Gamma distribution interpretation, we predicted that a Speed-Body Clock of 0.8 resulted in a speed Speed-Body Age of $e^{(4.8-0.43*0.8)}$=86.14 in InCHIANTI and 94.44 in BLSA (Fig. S8C&D).

Fig S8. Speed-Body Clock. A BLSA. B InCHIANTI. Speed-Body Age: C BLSA. D. InCHIANTI**.** The smaller the values, the worse the health is considered for both Speed-Body Clock and Speed-Body Age.

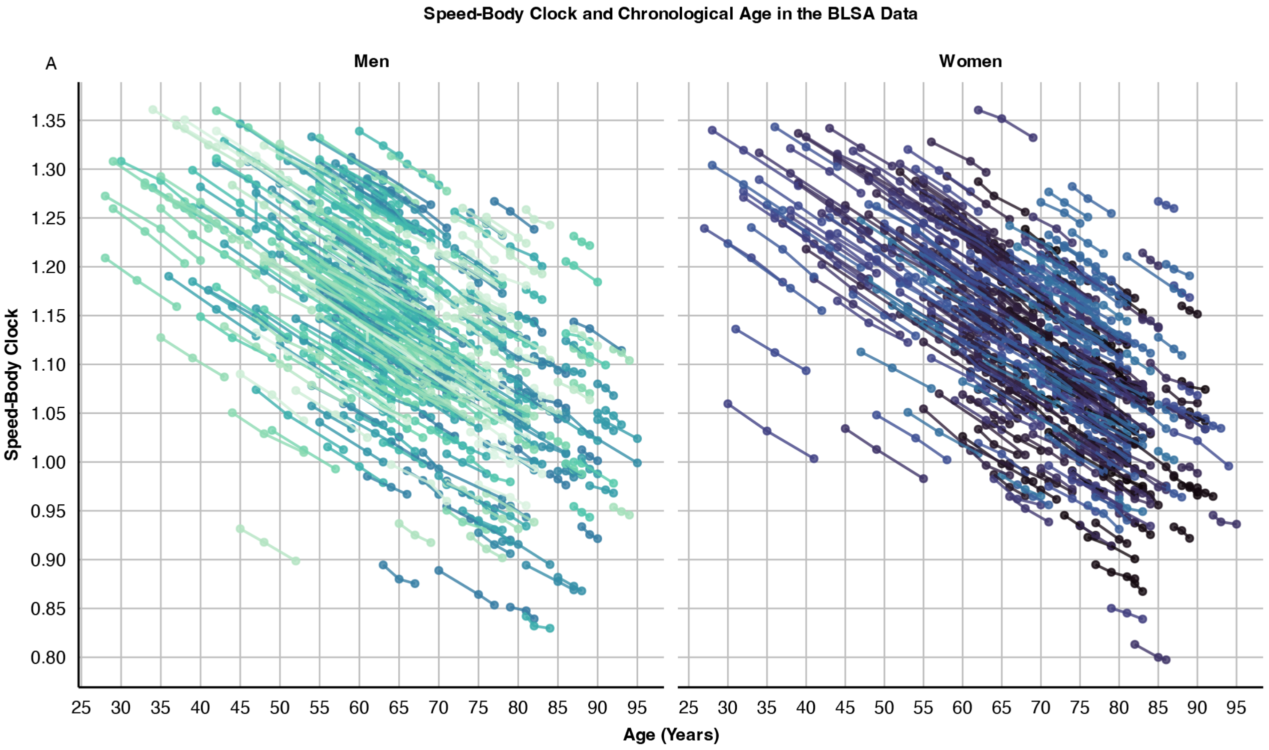

Speed-Body Clock and Chronological Age in InCHIANTI

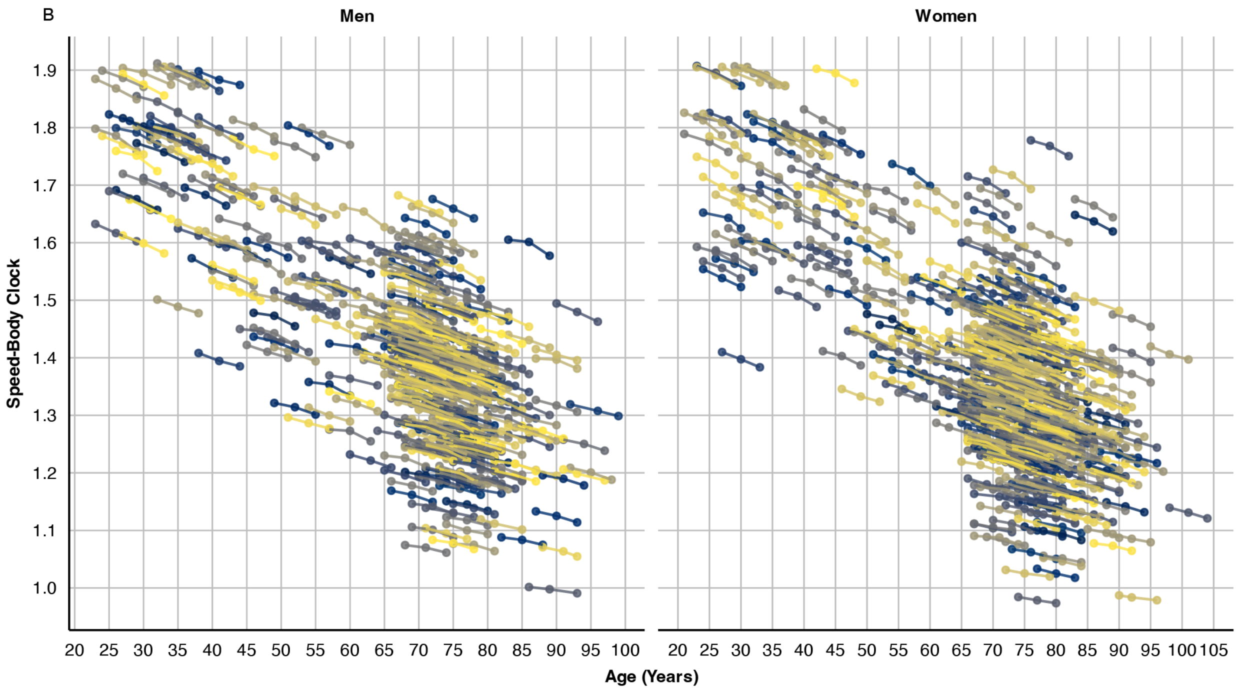

Speed-Body Age and Chronological Age in the BLSA Data

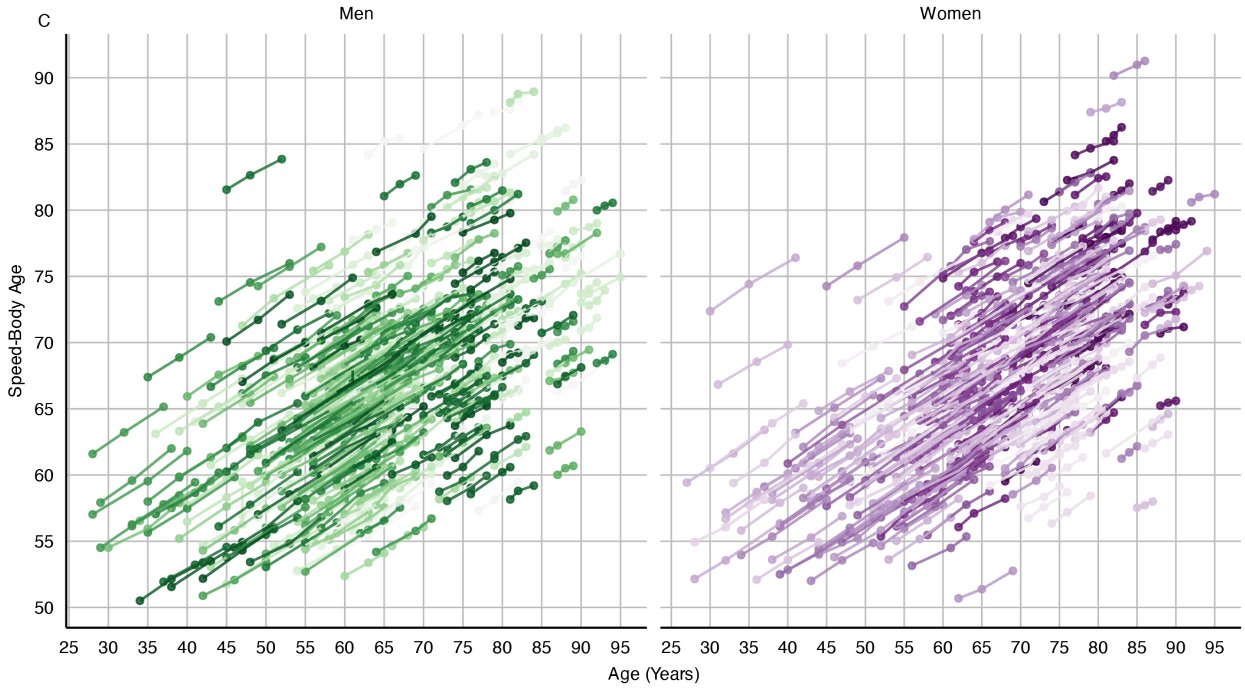

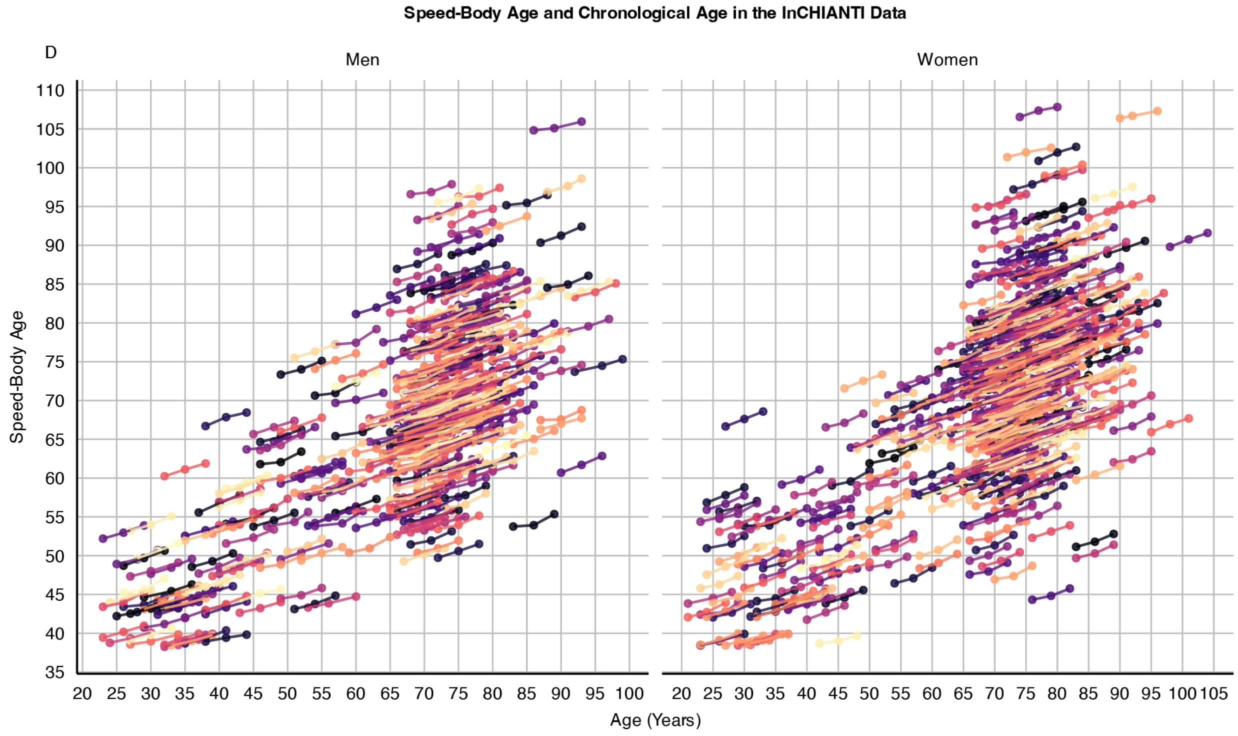

Disability-Body Clock and Disability-Body Age

Disability-Body Clock is incident of disability conditioned on total number of disability items measured and predicted by Body Clock. There is an increase in heterogenity of Disability-Body Clock with chronological age. Regressing chronological age over Disability-Body Clock resulted in the Disability-Body Age as rate of aging. The heterogenity of rate of aging increased with chronologicsal age (Fig. S9).

Fig S9. Disability-Body Clock and chronological age in A BLSA, B InCHIANTI data.

Disability-Body Age: C BLSA, D InCHIANTI.

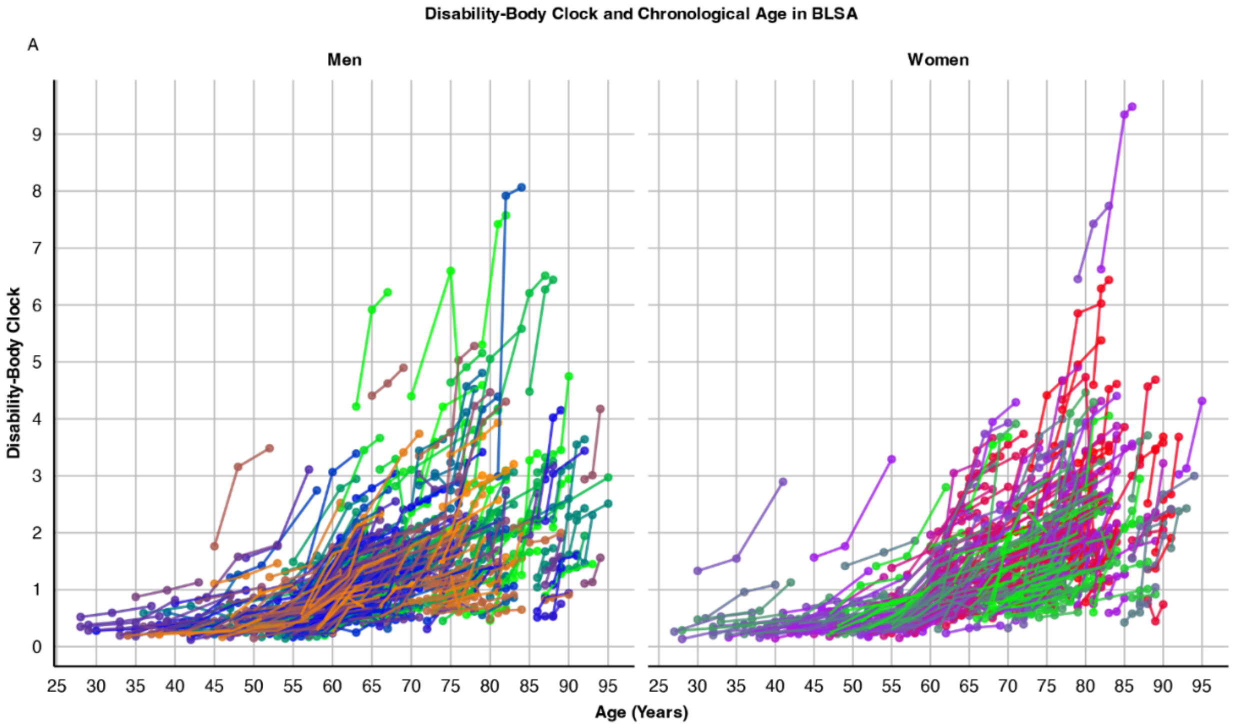

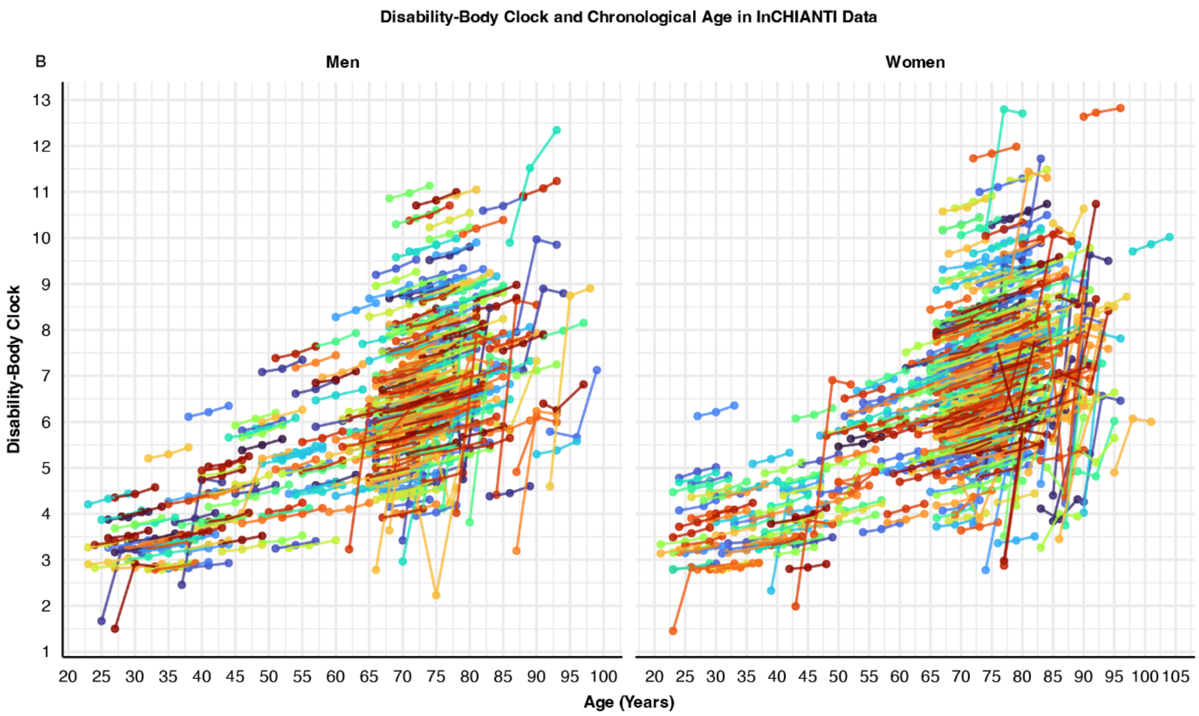
Disability-Body Clock and Chronological Age in InCHIANTI Data
B
Men
Women
Disability-Body Clock
Age (Years)

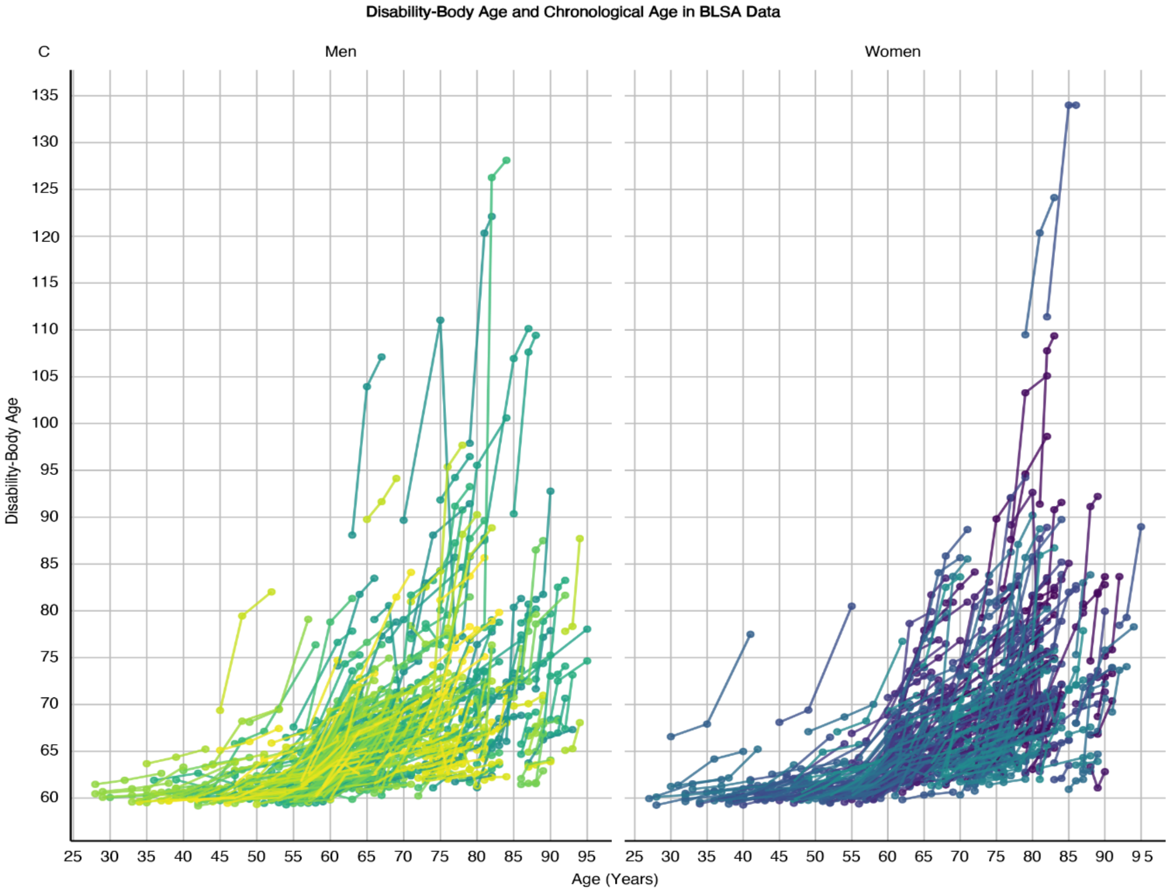
Disability-Body Age and Chronological Age in BLSA Data
C
Men
Women
Disability-Body Age
Age (Years)

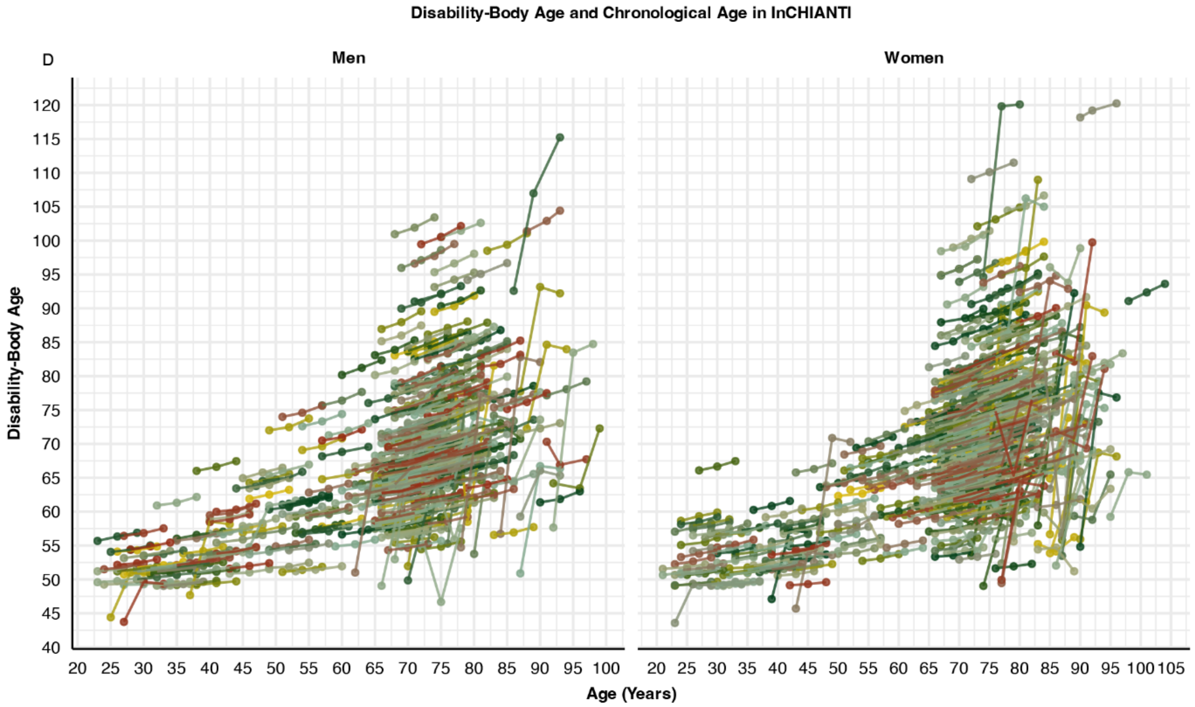

Body Clock versus Frailty Index

The association between the Body Clock and the Frailty Index (FI) reveals a strong correlation between the two approaches to health metrics (In InCHIANTI: r= 0.89, 95%CI 0.877 – 0.893; in NHNAES: r= 0.92, 95%CI: 0.915 – 0.918) . However, the Body Clock captures heterogeneity more effectively than FI. Specifically, for heterogeneous values of the Body Clock that serve as a proxy for the intrinsic clock and health entropy, corresponding FI values remain largely unchanged (Fig. S10).

Fig. S10. FI vs. the Body Clock: A in InCHIANTI data. B In NHANES data.

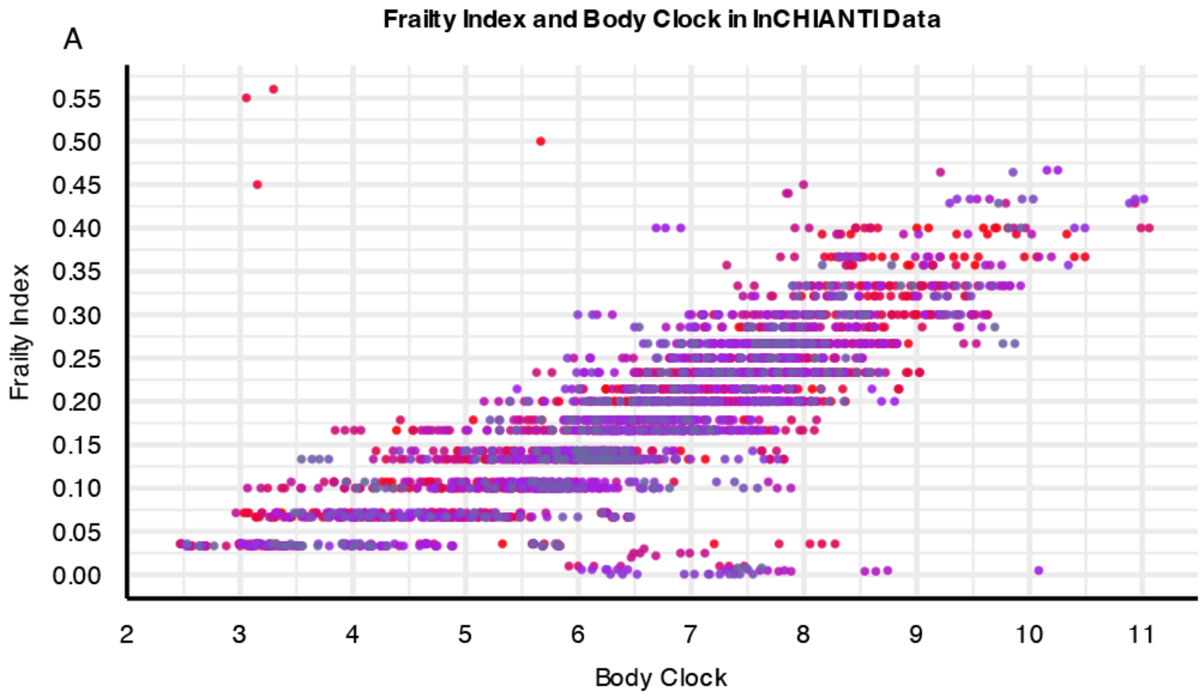

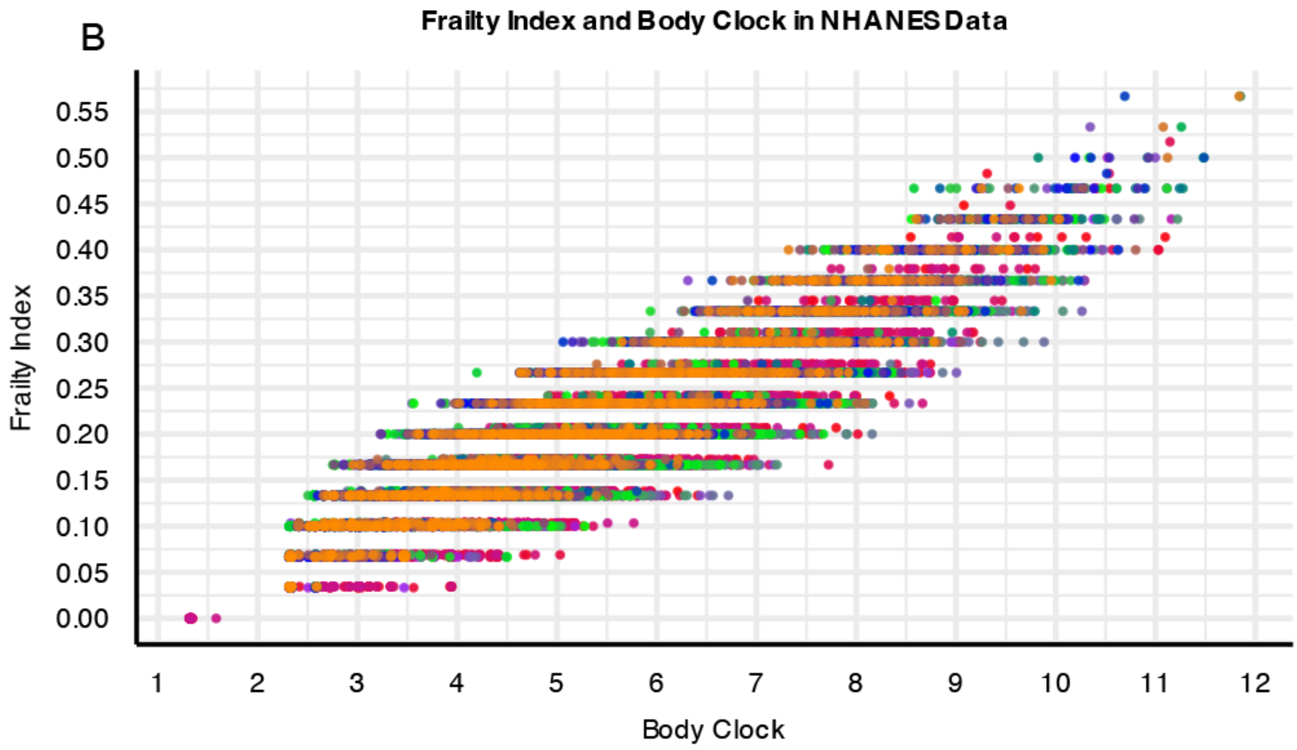

Body Clock vs FI for predicting outcomes

The Body Clock predicts binary SPPB<9, geriatric syndrome, and disability, superseding the FI score with more than 90% accuracy.

Fig. S11 Receiver Operating Characteristic (ROC) and Area Under the Curve (AUC) of the Body Clock and FI score in predicting binary outcomes in the InCHIANTI data as the replication of results in the BLSA data.

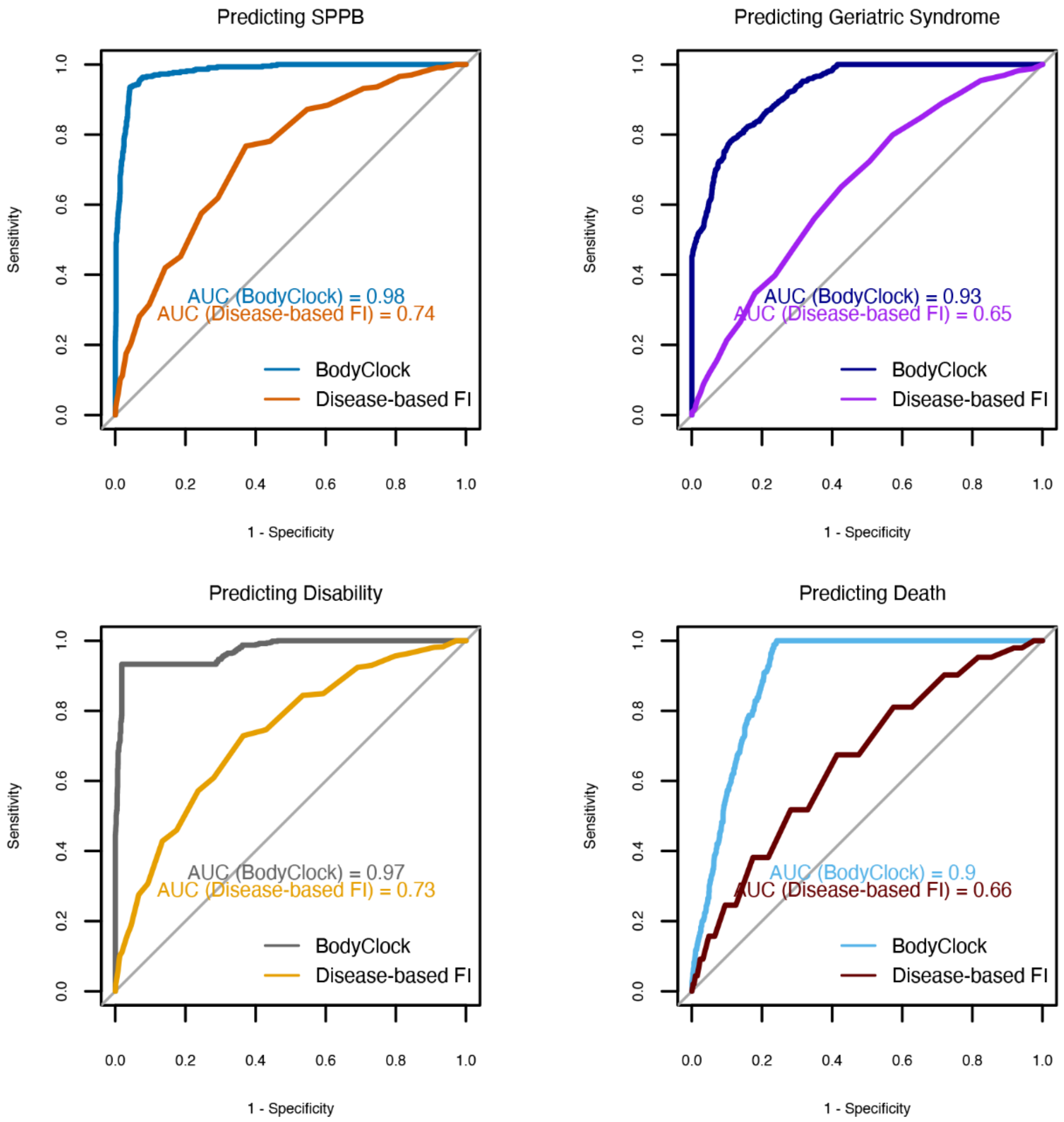

Replication of the entropy (full) model using NHANES data

The Body Clock and Body Age in more than 40,700 individuals were obtained from the NHANES data over 16 years. Both Body Clock and Body Age increase with chronological age (Fig S12). The distributions of the number of systems contributing to the Body Clock suggest that morbidity in more than 5 organs becomes dominant after age 50 and older. However, some older adults with low Body Clock values exhibit bodily system resilience. Body Age versus chronological age shows that rate of aging surpasses the reported maximum chronological in humans (Fig. S12)

Fig. S12. The Body Clock and Chronological Age in NHANES Data. A Predicted Body Clock with chronological age. B Predicted Body Age with chronological age. The number of systems contributing to Body Clock or Body Age are depicted using different colors.

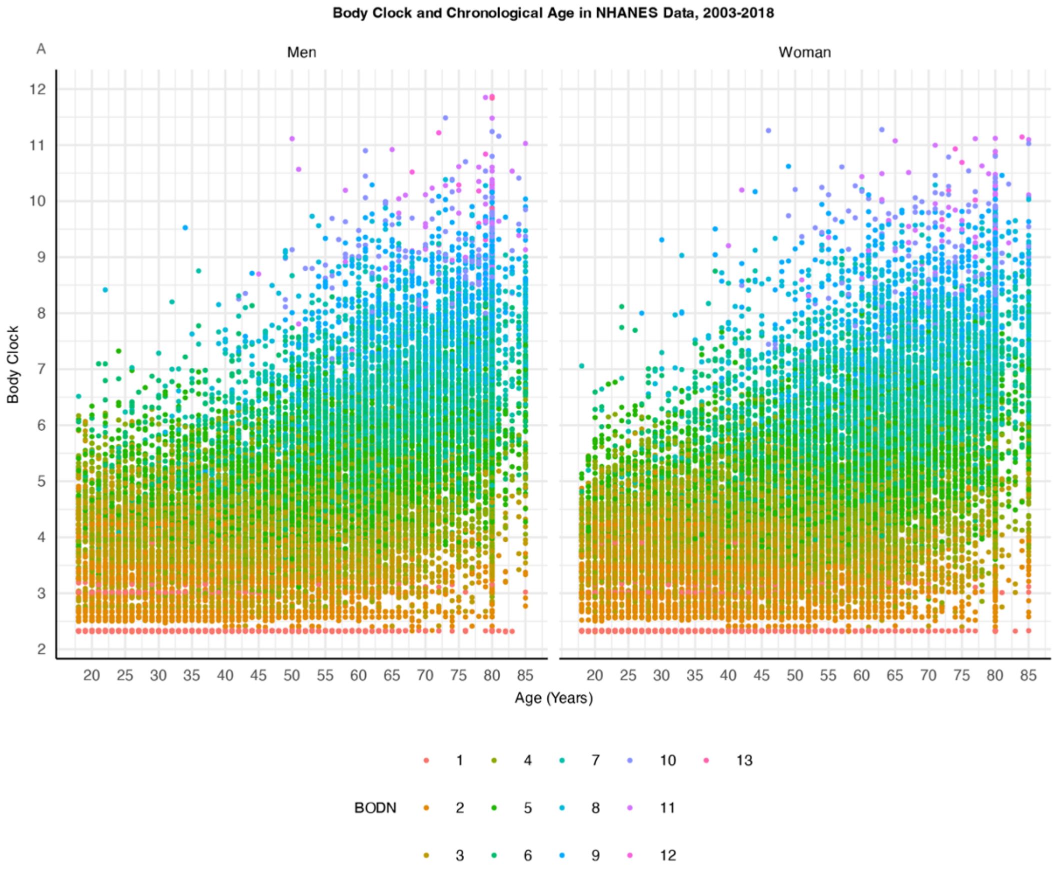

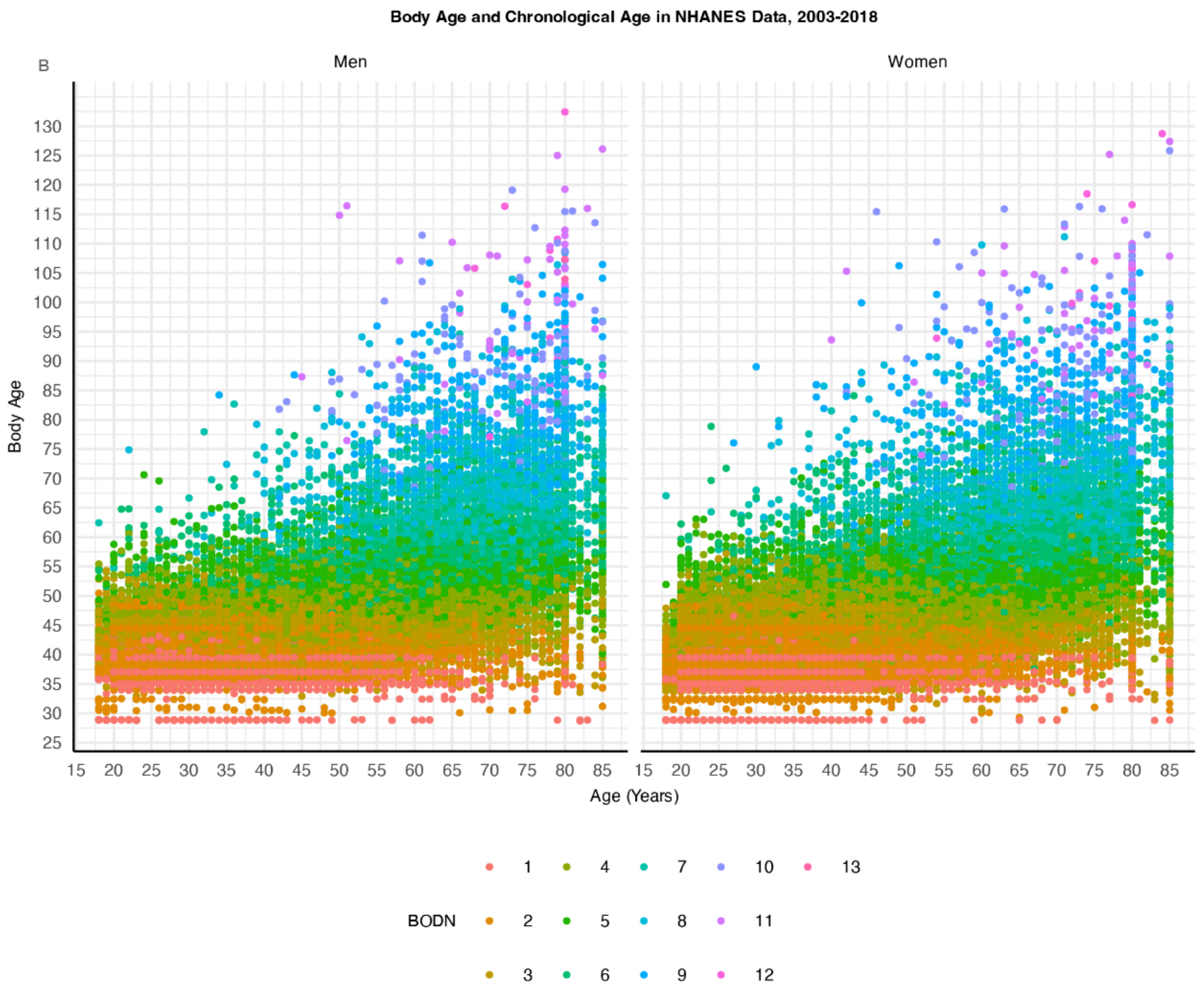

The Body Clock Predicts Mortality (death) in NHANES data

The NHANES data reveals that the Body Clcoksignificantly predicts mortality. The ROC (receiver operating characteristic curve) analysis and area under the curve (AUC) shows the Body Clock performs better than the FI score in predicting mortality (AUC=0.8).

Fig. S13. The Body Clock predicts death (mortality) in NHANES data.

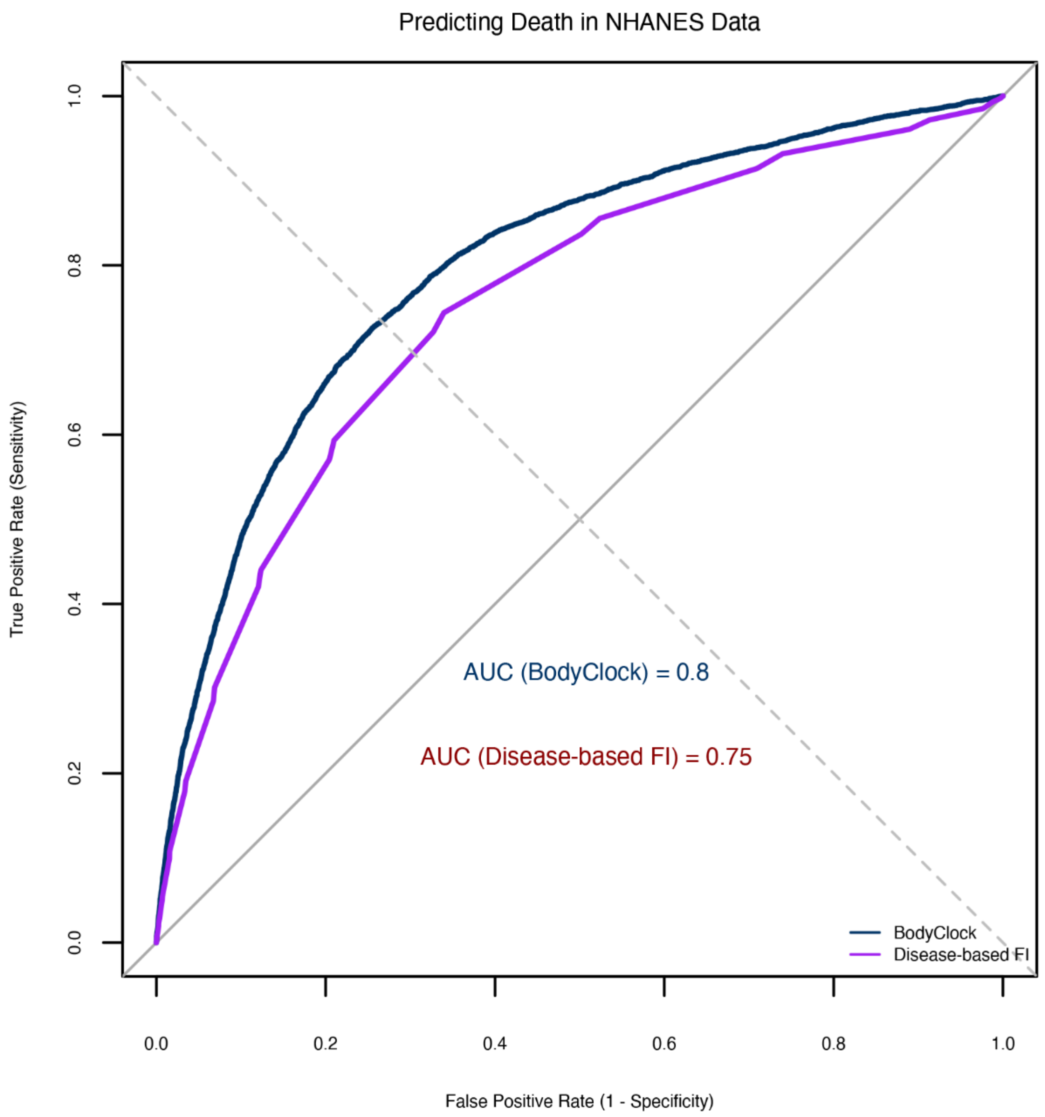

Out-of-Data Model Validations to Predict BODN

To assess the robustness of the model to predict BODN, we used the time-1 full model based on BLSA data to predict BODN in the InCHIANTI and NHANES data as out-of-sample validations. Additionally, we used "posterior predictive checking" [11] implemented in the brms software

package to compare the posterior predictive density of simulated data to density estimates of the observed data (in-sample evaluation of BLSA) or predict new data (InCHIANTI data; out-of-sample validation) (Fig. S14).

Fig. S14. Out-of-data validation using BLSA full model to predict BODN. A in the InCHIANTI data. B NHANES data 2003-2018)**.**

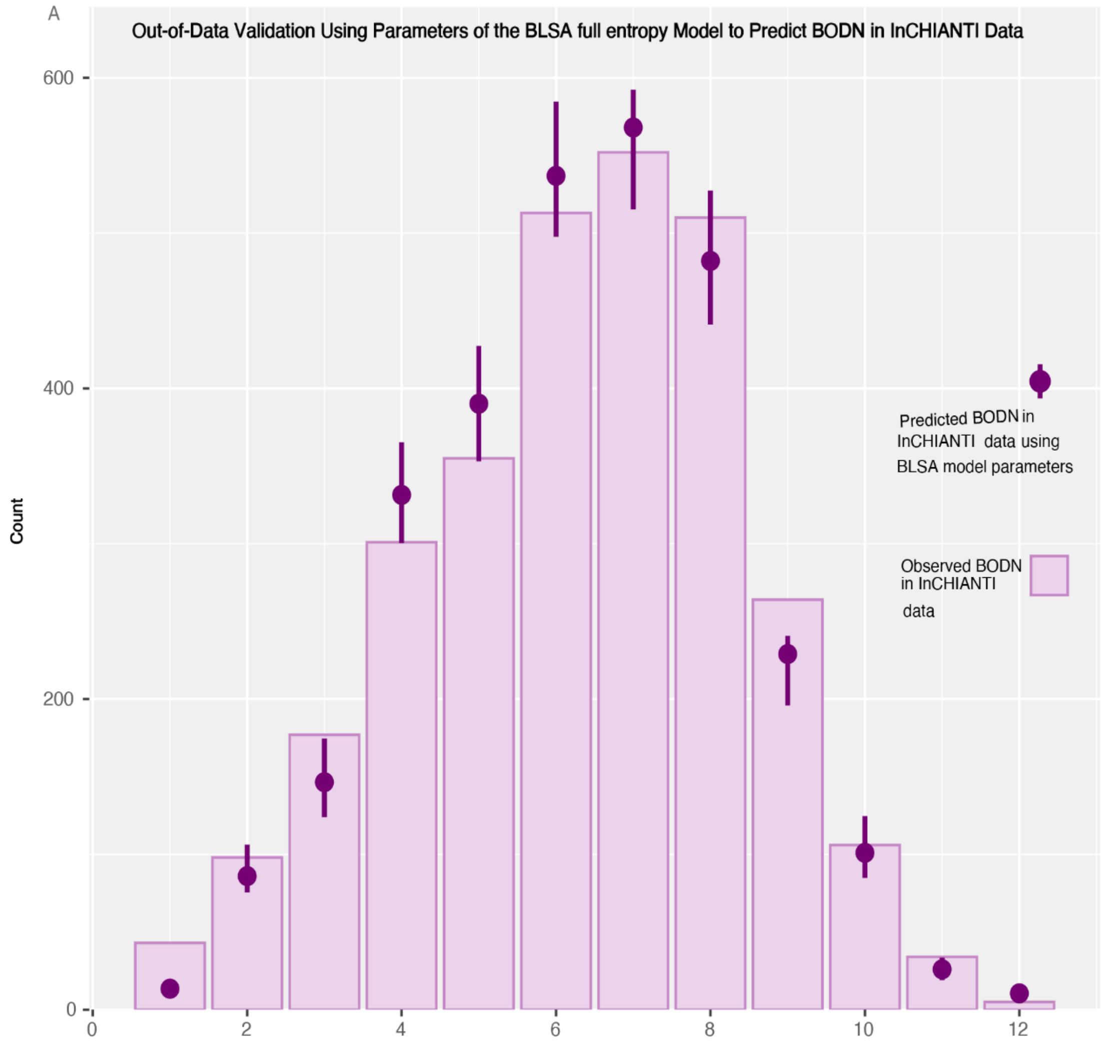

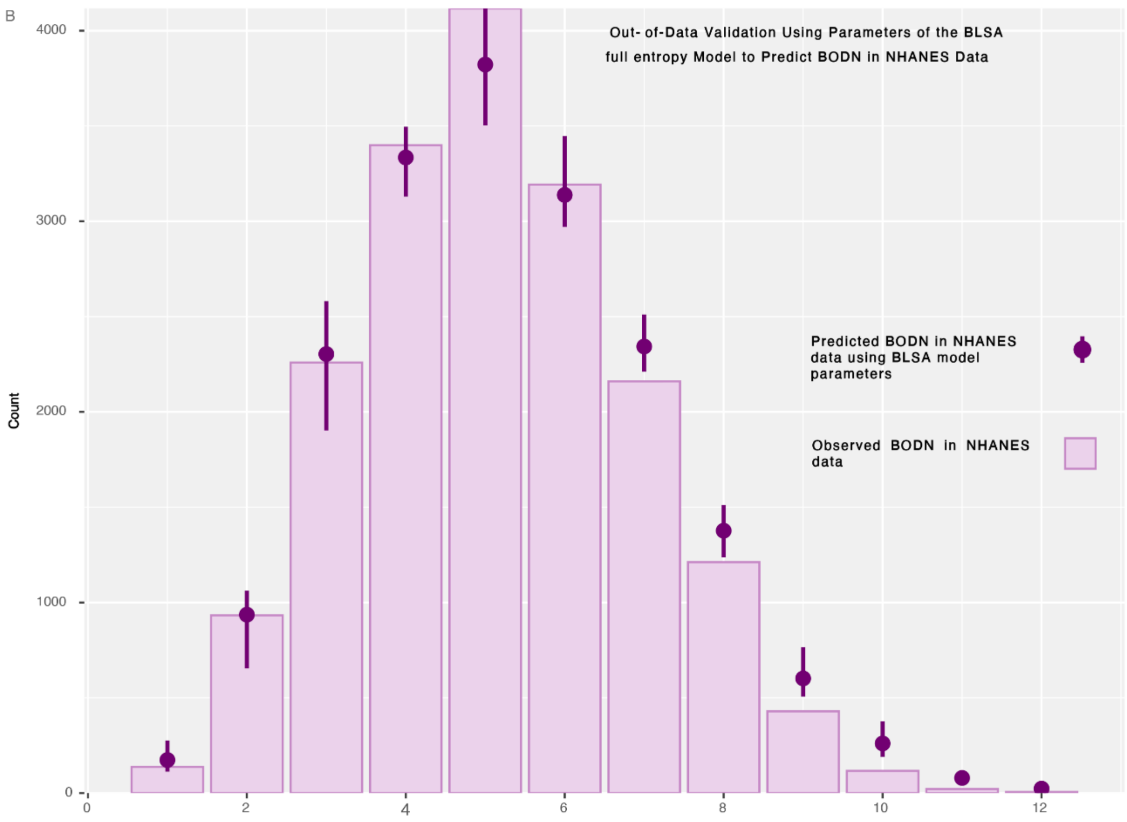

B
Out- of-Data Validation Using Parameters of the BLSA
full entropy Model to Predict BODN in NHANES Data
Predicted BODN in NHANES data using BLSA model parameters
Observed BODN in NHANES data
Count
4000
3000
2000
1000
0
0
2
4
6
8
10
12

References

1 Kuo, P. L. *et al.* A roadmap to build a phenotypic metric of ageing: insights from the Baltimore Longitudinal Study of Aging. *J Intern Med* **287**, 373-394 (2020). https://doi.org/10.1111/joim.13024

2 Ferrucci, L. *et al.* Subsystems contributing to the decline in ability to walk: bridging the gap between epidemiology and geriatric practice in the InCHIANTI study. *J Am Geriatr Soc* **48**, 1618-1625 (2000). https://doi.org/10.1111/j.1532-5415.2000.tb03873.x

3 *Centers for Disease Control and Prevention. National Health and Nutrition Examination Survey*, <https://www.cdc.gov/nchs/nhanes/index.htm> (2003-2018).

4 Cichoz-Lach, H. *et al.* The BARD score and the NAFLD fibrosis score in the assessment of advanced liver fibrosis in nonalcoholic fatty liver disease. *Med Sci Monit* **18**, CR735-740 (2012).

5 Neder, J. A., Berton, D. C. & O'Donnell, D. E. Calculating the statistical limits of normal and Z-scores for pulmonary function tests. *J Bras Pneumol* **48**, e20220182 (2022). https://doi.org/10.36416/1806-3756/e20220182

6 Vaz Fragoso, C. A., Van Ness, P. H. & McAvay, G. J. FEV(1) as a Standalone Spirometric Predictor and the Attributable Fraction for Death in Older Persons. *Respir Care* **65**, 217-226 (2020). https://doi.org/10.4187/respcare.07012

7 Biondi, B., Cappola, A. R. & Cooper, D. S. Subclinical Hypothyroidism: A Review. *JAMA* **322**, 153-160 (2019). https://doi.org/10.1001/jama.2019.9052

8 McElreath, R. *Statistical Rethinking: A Bayesian Course with Examples in R and Stan*. (Chapman and Hall/CRC, 2016).

9 Gelman, A., Carlin, J., Stern, H., Dunson, D., Vehtari, A. & Rubin, D. *Bayesian Data Analysis*. (2014).

10 Vehtari, A., Gelman, A. & Gabry, J. Practical bayesian model evaluation using leave-one-out cross-validation and WAIC. *Statistics and Computing* **27**, 1413-1432 (2017).

11 Gabry, J., Simpson,D., Vehtari, A., Betancourt, M., Gelman, A. Visualization in Bayesian workflow. *Journal of the Royal Statistical Society Series A* **182**, 389-402 (2019).

12 Bürkner, P.C., Vuorre, M. Ordinal Regression Models in Psychology: A Tutorial. *Advances in Methods and Practices in Psychological Science* **2**, 77-101 (2019).

13 Bürkner, P. C., Charpentier., E. Modelling monotonic effects of ordinal predictors in Bayesian regression models. *Br J Math Stat Psychol* **73**, 420-451 (2020). https://doi.org/10.1111/bmsp.12195

14 Bürkner, P. C. Advanced Bayesian Multilevel Modeling with the R Package brms. *The R Journal* **10**, 395–411 (2018).

15 Carpenter, B., Gelman, A., Hoffman, M.D., Lee, D., Goodrich, B., Betancourt, M., Riddell, A. Stan: A probabilistic programming language. *Journal of Statistical Software 76.* (2017).

16 Vehtari, A., Gelman, A., Simpson, D., Carpenter, B., Bürkner, P.C. Rank-normalization, folding, and localization:An improved R-hat for assessing convergence of MCMC. *Bayesian analysis*, 1-28 (2020).

17 Salimi, S. & Hamlyn, J. M. COVID-19 and Crosstalk With the Hallmarks of Aging. *J Gerontol A Biol Sci Med Sci* **75**, e34-e41 (2020). https://doi.org/10.1093/gerona/glaa149

Table S2. Health Systems Questions

**Cardiovascular system**		
cv_ihd1: In the past year have you had any chest pain that took more than 5 minutes but less than 30 minutes?	Yes	No
cv_ami_1: In the past year have you had any chest pain that took more than 30 minutes?	Yes	No
cv_ihd1_2: In the past year have you ever told you had ischemic heart disease?	Yes	No
cv-ami_2: In the past year have you had acute myocardial infarction?	Yes	No
cv_ihd_3_3: In the past year have you used any medications for ischemic heart disease?	Yes	No
cv_htn_1: What is your most recent systolic blood pressure? (mmHg)	Yes	No
cv_htn_2: What is your most recent diastolic blood pressure? (mmHg)	Yes	No
cv_htn_3: In the past year have you told you have hypertension?	Yes	No
cv_htn4: In the past year have you ever used medication for high blood	Yes	No

pressure?		
chf_1: Have you had shortness of breath at rest without having asthma?	Yes	No
chf_2: Have you had worsening shortness of breath when lying flat?	Yes	No
chf_3: Have you had increased Need to Urinate at Night?	Yes	No
chf_4: Have you had history of congestive heart failure?	Yes	No
chf_5: Have you used medication for get rid of water?	Yes	No
chf_6: Have you used digoxin?	Yes	No
chf_7: Have you had used Spironolactone for your heart?	Yes	No
chf_8: Have you used Furosemide (Lasix) as water pill?	Yes	No
chf_9: Have you used Diltiazem?	Yes	No
chf_10: Have you used verapamil?	Yes	No
chf_11: Have you had ejection fraction >=40 in echocardiography?	Yes	No
chf_12: Have you had ejection fraction <40 in	Yes	No

echocardiography?		
pad_1: Have you had muscle pain, cramping, or heaviness in the legs or buttocks during physical activities such as walking or climbing stairs, which usually resolves with rest?	Yes	No
pad_2: Have you had hair loss or slower hair growth on the affected limb for peripheral artery disease?	Yes	No
pad_3: Have you had paleness of the affected limb for peripheral artery disease?	Yes	No
pad_4: Have ever been told by a doctor that you have peripheral artery disease?	Yes	No
pad_5: Do you have Ankle brachial index <0.9?	Yes	No
arr_1: Have you ever told you have irregular heartbeat?	Yes	No
arr_2: Have you ever had heartbeat less than 60?	Yes	No
arr_3: Have you ever had heartbeat more than 120?	Yes	No
arr_4: Have you ever told you need pacemaker?	Yes	No
arr_5: Have you ever told you need to take blood thinner because of irregular	Yes	No

heartbeat?

Stroke

st_1: Have you ever had the numbness, or weakness in the arm, leg, or face, on one side of the body that took less than 24 hours?	Yes	No
st_2: Have you ever been told by doctor that you had transient ischemic attack?	Yes	No
st_3: Have you ever had history of stroke or elongated sudden numbness, loss of speech with no paralysis or limb weakness?	Yes	No
st_4: Paralysis or paresis in either limb or any sudden long-lasting (more than 24 hours) paralysis in face?	Yes	No

Kidney

ren_1: Have you ever told by doctor that you have chronic kidney disease?	Yes	No
ren_2: Have you ever measured serum creatinine in your routine annual checkup?	Yes	No

ren_3: If yes what is the last time serum creatinine? (mg/dL)

ren_4: Have you been on kidney dialysis in past six months?	Yes	No
ren_5: Is your eGFR >90?	Yes	No
ren_6: Is your eGFR<=90 and eGFR >=60?	Yes	No
ren_7: Is your eGFR<=59 and eGFR>=45?	Yes	No
ren_8: Is your eGFR <=44 and eGFR>=30?	Yes	No
ren_9: Is your <=29 or you are on dialysis?	Yes	No

Metabolic - Diabetes

dm_1: Have you ever told by doctor you have diabetes after age 20 years old?	Yes	No
dm_2: Did your diabetes started when you were a child?	Yes	No
dm_3: Have you used insulin for controlling of your diabetes?	Yes	No
dm_4: Have you used any other medications than insulin for diabetes?	Yes	No

Metabolic - Hyperlipidemia

lip_1: Have your doctor ever told you that you have high bad cholesterol?	Yes	No

lip_2: Have your doctor ever told you that you have high triglyceride?	Yes	No
lip_3: What was your blood LDL (low density cholesterol) cholesterol levels last time in mg/dL?		
lip_4: What was your blood triglyceride (mg/dL) levels last time in mg/dL?		
lip_5: What was your high-density cholesterol (HDL) last time in mg/dL?		
lip_6: Have you ever used any medication for increased cholesterol level?	Yes	No
lip_7: Have you ever used any medication for increased triglyceride level?	Yes	No

Liver

liv_1: Have you told by a doctor that you have hepatitis?	Yes	No
liv_2: Have you told by a doctor that you have liver fibrosis?	Yes	No
liv_3: Have you told by a doctor that you have liver cirrhosis?	Yes	No
liv_4: What was the level of your ALT/SGPT in U/L last time measured?		

liv_5: What was the level of your AST/SGOT in U/L last time measured?

liv_6: What was the level of your platelet (109/L) last time measured?

liv_7: What was level of fibrinogen last time measured?

liv_8: What was the level of serum/plasma albumin in g/dL?

liv_9: What is your weight in Kg?

liv_10: What is your height in centimeter?

Gastrointestinal system

gi_1: Have you ever been told by a doctor that you have gastroesophageal reflux?	Yes	No
gi_2: Have you ever been told by a doctor that you have gastritis?	Yes	No
gi_3: Have you ever had heartburn in past year?	Yes	No
gi_4: Have you ever had heartburn in the past year?	Yes	No
gi_5: Have you ever experienced regurgitation in the past year?	Yes	No

gi_6: Have you ever had difficulty swallowing (dysphagia) in the past year?	Yes	No
gi_7: Have you experienced hoarseness or a sore throat in the past year?	Yes	No
gi_8: Have you felt bloated in the past year?	Yes	No
gi_9: Have you had frequent belching in the past year?	Yes	No
gi_10: Have you had abdominal pain or discomfort in the past year?	Yes	No
gi_11: Have you experienced indigestion in the past year?	Yes	No
gi_12: Have you had dark stools in the past year?	Yes	No
gi_13: Have you had excessive burping in the past year?	Yes	No
gi_14: Have you felt fatigued due to stomach or intestine symptoms in the past year?	Yes	No
gi_15: Have you felt stomach pain before meal?	Yes	No
gi_16: Have you felt stomach pain after meal?	Yes	No

gi_17: Have you been tested positive for h-pylori?	Yes	No
gi_18: Have you ever noticed black or tarry stools in the past year?	Yes	No
gi_19: Have you ever experienced coffee ground vomit in the past year?	Yes	No
gi_20: Have you ever used any medication for stomach problem or indigestion?	Yes	No

Respiratory

res_1: Have you ever been told by a doctor that you have a chronic obstructive pulmonary disease?	Yes	No
res_2: Have you ever been told by a doctor that you have a chronic bronchitis?	Yes	No
res_3: Have you ever been told by a doctor that you have emphysema?	Yes	No
res_4: Have you had phlegm on most days for 3 consecutive months or more during the year?	Yes	No
res_5: Have you done respirometry within past year?	Yes	No
res_6: What was the values for FEV1?		

res_7: What was the value for FVC?		
res_8: Was FEV1≥ 2.00 L?	Yes	No
res_9: Was 1.99 ≤ FEV ≤ 1.50 L?	Yes	No
res_10: Have you ever been prescribed any medication for chronic obstructive pulmonary disease (COPD)?	Yes	No

Thyroid

thyr_1: Have you ever been told by a doctor that you have hypothyroidism?	Yes	No
thyr_2: Have you ever been prescribed medication for hypothyroidism?	Yes	No
thyr_3: What was the levels of thyroid stimulating hormone (TSH) mIU/L last time measured?	Yes	No
thyr_4: Have you taken any medication for low thyroid function in the past six months?	Yes	No

Hematopoietic system

he_1: Have you ever been told you have anemia by a doctor?	Yes	No
he_2: What was your hemoglobin level (Hb		

g/dL) last time?		
he_3: Have you ever been told you have low platelet by doctor?	Yes	No
he_4: Have you ever been told you are at risk of bleeding because of low platelet count?	Yes	No
he_5: What was your platelet counts in the last time (109/L)?		
he_6: Have you ever been told by doctor you have low white blood cell (WBC)?	Yes	No
he_7: Have you ever been told by a doctor that you are at risk of infection because of low white blood cells (WBC)?	Yes	No
he_8: What was your white blood cells counts last time (103/µL)?		

Oral health

or_1: Have you had red, swollen gums in the past six months?	Yes	No
or_2: Have you experienced bleeding gums during brushing or flossing in the past six months?	Yes	No
or_3: Have you had persistent bad breath	Yes	No

(halitosis) in the past six months?		
or_4: Have you noticed soft, tender gums in the past six months?	Yes	No
or_5: Have your gums had a change in texture (e.g., smooth or spongy) in the past six months?	Yes	No
or_6: Have you had deepening pockets between your gums and teeth in the past six months?	Yes	No
or_7: Have you noticed loose or shifting teeth in the past six months?	Yes	No
or_8: Have you experienced persistent pain or discomfort in your gums or teeth in the past six months?	Yes	No
or_9: Have you had pus around your teeth and gums in the past six months?	Yes	No
or_10: Have you had changes in how your teeth fit together (bite) in the past six months?	Yes	No
or_11: Have you experienced swollen, painful gums in the past six months?	Yes	No
or_12: Have you noticed a foul taste in your mouth in	Yes	No

the past six months?		
or_13: Do you have >50% tooth loss due to periodontitis or gingivitis?	Yes	No

Osteoarthritis

oa_1: Have you ever been told by a doctor that you have osteoarthritis?	Yes	No
oa_2: Have you had Pain in the affected joints during or after movement that worsens with activity and improves with rest?	Yes	No
oa_3: Have you ever experienced Joint stiffness after periods of inactivity, particularly noticeable in the morning?	Yes	No
oa_4: Have you noticed Swelling or tenderness in the affected joints?	Yes	No
oa_5: Have you experienced Reduced range of motion in any of your joints?	Yes	No
oa_6: Have you felt A grating sensation or sound (crepitus) when using the affected joint?	Yes	No
oa_7: Have you observed any Joint deformities or noticeable changes in the shape of your joints?	Yes	No

oa_8: Have you developed any Bone spurs around your joints that cause discomfort?	Yes	No
oa_9: Have you experienced Muscle weakness around the affected joints due to disuse?	Yes	No
oa_10: Have you felt Chronic fatigue due to ongoing joint pain and discomfort?	Yes	No
oa_11: Do you find that your symptoms worsen during cold weather or changes in humidity?	Yes	No

Osteoporosis

ost_1: Have you ever been told by a doctor that you have osteopenia?	Yes	No
ost_2: Have you ever been told by a doctor that you have osteoporosis?	Yes	No
ost_3: Have you done dual-energy X-ray absorptiometry (DEXA) scans to assess your bone mineral density (BMD)?	Yes	No
ost_4: What was your standardized bone mineral density (BMD) value?		
ost_5: Was the standardized bone mineral	Yes	No

density (BMD) value was <-1.0?		
ost_6: Was the standardized bone mineral density (BMD) value was <-2.5?	Yes	No

Gout

gout_1: Have you ever been told by a doctor that you have gout?	Yes	No
gout_2: Have you measured serum/plasma uric acid (mg/dL) in past six months?	Yes	No
gout_3: What was the levels of uric acid in your blood (mg/dL)?		
gout_4: Have you been told by a doctor that you have high levels of uric acid in your blood?	Yes	No
gout_5: Have you taken allopurinol in the past six months?	Yes	No
gout_6: Have you taken colchicine in the past six months?	Yes	No

Sensory system

sens_1: Have you had normal hearing in past six months?	Yes	No

sens_2: Have you ever experienced hearing decreased hearing ability past six months?	Yes	No
sens_3: Have you had fair hearing ability with hearing aid in past six months?	Yes	No
sens_4: Have you had poor hearing with a hearing aid in past six months?	Yes	No
eye_1: Have you had eye problem in past six months?	Yes	No
eye_2: Have you had only cataract or cataract surgery in past six months (not glaucoma or macular degeneration)?	Yes	No
eye_3: Have you ever told by a doctor that you have high pressure in your eye or diagnosed with only glaucoma?	Yes	No
eye_4: Have you ever told by a doctor that you have only macular degeneration?	Yes	No
eye_5: Have you ever told you have both glaucoma and macular degeneration?	Yes	No

Depression

dep_1: During past week I was bothered by things that	Yes	No

usually don’t bother me.		
dep_2: I did not feel like eating; my appetite was poor	Yes	No
dep_3: I felt that I could not shake off the blues even with help from my family and friends	Yes	No
dep_4: I felt that I was just as good as other people	Yes	No
dep_5: I had trouble keeping my mind on what I was doing	Yes	No
dep_6: I felt depressed	Yes	No
dep_7: I felt that everything I did was an effort	Yes	No
dep_8: I felt hopeful about the future	Yes	No
dep_9: I thought my life had been a failure	Yes	No
dep_10: I felt fearful	Yes	No
dep_11: My sleep was restless	Yes	No
dep_12: I was happy	Yes	No
dep_13: I talked less than usual	Yes	No
dep_14: I felt lonely	Yes	No
dep_15: People were unfriendly	Yes	No

dep_16: I enjoyed life	Yes	No
dep_17: I had crying spells	Yes	No
dep_18: I felt sad	Yes	No
dep_19: I felt that people disliked me	Yes	No
dep_20: I could not get "going"	Yes	No

Parkinson

Park_1: Have you been told by a doctor that you have Parkinson?	Yes	No
Park_2: Have you been using medication for Parkinson disease?	Yes	No

Hoehn and Yahr Scale

HY_1: Positive diagnosis of Parkinson and unilateral involvement only in past six months?	Yes	No
HY_2: Positive diagnosis of Parkinson and unilateral and axial involvement?	Yes	No
HY_3: Positive diagnosis of Parkinson and bilateral involvement without impairment of balance?	Yes	No
HY_4: Positive diagnosis of Parkinson and mild bilateral disease with recovery on pull test?	Yes	No

HY_5: Positive diagnosis of Parkinson and mild to moderate bilateral disease; some postural instability; physically independent?	Yes	No
HY_6: Positive diagnosis of Parkinson and severe disability; still able to walk or stand unassisted?	Yes	No
HY_7: Positive diagnosis of Parkinson and wheelchair bound or bedridden unless aided?	Yes	No

Cognition

cog_1: Have you been told by a doctor that you have cognitive impairment in past six months?	Yes	No
cog_2: Have you been examined for mini mental state and scored less than 24?	Yes	No
cog_3: What was the score for your Mini Mental State in past six months?		
cog_med_1: Have you taken any medication for cognitive decline in past six months?	Yes	No

Cancer

can_1: Have you been diagnosed with malignant cancer (Except basal cell	Yes	No

carcinoma of skin)?		
can_2: Have you been told by the doctor that you have lung cancer?	Yes	No
can_3: Have you been told by the doctor that you have breast cancer?	Yes	No
can_4: Have you been told by the doctor that you have prostate cancer?	Yes	No
can_5: Have you been told by the doctor that you have stomach cancer?	Yes	No
can_6: Have you been told by the doctor that you have pancreatic cancer?	Yes	No
can_7: Have you been told by the doctor that you have thyroid cancer?	Yes	No
can_8: Have you been told by the doctor that you have ovary cancer?	Yes	No
can_9: Have you been told by the doctor that you have leukemia?	Yes	No
can_10: Have you been told by the doctor that you have lymphoma?	Yes	No
can_11: Have you been told by the doctor that you have renal cancer?	Yes	No
can_12: Have you been told by the doctor that you	Yes	No

have liver cancer?		
can_13: Have you been told by the doctor that you have brain cancer?	Yes	No
can_14: Have you been told by the doctor that you have combination of several cancers?	Yes	No
can_15: Have you been told by the doctor that you have squamous cell carcinoma?	Yes	No
can_16: Have you been told by the doctor that you have colon cancer?	Yes	No
can_17: Have you been told by the doctor that you have bone cancer?	Yes	No
can_18: Have you been told by the doctor that you have cartilage cancer?	Yes	No

Table S6A. Time-1 Single Disease Models Predicting Longitudinal BODN in BLSA Data

Disease	Posterior Coef	SE	2.5QI	97.5QI
HTN	1.56	0.21	1.15	1.97
HTN[L2]	1.36	0.18	1.00	1.71
HTN[L3]	0.20	0.03	0.15	0.26
IHD	1.44	0.13	1.17	1.70
IHD[L2]	0.56	0.05	0.46	0.66
IHD[L3]	0.88	0.08	0.71	1.04
CHF	1.76	0.18	1.41	2.11
CHF[L2]	0.60	0.06	0.48	0.72
CHF[L3]	1.16	0.12	0.93	1.39
Arr	0.70	0.14	0.46	0.99
Arr[L2]	0.12	0.02	0.08	0.17
Arr[L3]	0.13	0.03	0.09	0.19
Arr[L4]	0.16	0.03	0.11	0.23
Arr[L5]	0.11	0.02	0.07	0.15
Arr[L6]	0.18	0.04	0.12	0.25
PAD	3.43	0.70	2.05	4.80
Stroke	1.61	0.23	1.17	2.07
Stroke[L2]	0.68	0.10	0.49	0.87
Stroke[L3]	0.18	0.03	0.13	0.23
Stroke[L4]	0.76	0.11	0.55	0.97
CKD	2.47	0.38	1.80	3.29
CKD[L2]	0.96	0.15	0.70	1.28
CKD[L3]	0.42	0.06	0.31	0.56
CKD[L4]	0.37	0.06	0.27	0.49
CKD[L5]	0.72	0.11	0.52	0.95
DM	1.03	0.18	0.71	1.42
DM[L2]	0.19	0.03	0.13	0.26
DM[L3]	0.07	0.01	0.05	0.10
DM[L4]	0.28	0.05	0.19	0.38
DM[L5]	0.12	0.02	0.09	0.17
DM[L6]	0.36	0.06	0.25	0.50
Lipid	0.35	0.21	-0.05	0.76
Lipid[L2]	0.16	0.10	-0.02	0.36
Lipid[L3]	0.19	0.11	-0.03	0.40
Liver	1.27	0.26	0.83	1.87
Liver[L2]	0.51	0.10	0.33	0.75
Liver[L3]	0.42	0.09	0.27	0.62
Liver[L4]	0.34	0.07	0.22	0.50
GID	1.57	0.18	1.22	1.92
GID[L2]	1.22	0.14	0.95	1.50
GID[L3]	0.35	0.04	0.27	0.42
COPD	2.75	0.45	1.92	3.65
COPD[L2]	1.93	0.32	1.34	2.56
COPD[L3]	0.83	0.14	0.58	1.10
Asthma	2.68	0.54	1.62	3.75

Hypoth	1.11	0.14	0.83	1.39
Hypoth[L2]	0.68	0.09	0.51	0.85
Hypoth[L3]	0.22	0.03	0.17	0.28
Hypoth[L4]	0.21	0.03	0.16	0.26
Hyperth	2.60	1.20	0.23	4.97
Anemia	1.42	0.33	0.84	2.13
Anemia[L2]	1.08	0.25	0.64	1.62
Anemia[L3]	0.34	0.08	0.20	0.51
Thrombocytopenia	0.66	0.64	-0.59	1.92
WBC	1.52	0.70	0.26	2.91
WBC[L2]	0.36	0.17	0.06	0.70
WBC[L3]	1.16	0.53	0.20	2.21
OA	1.40	0.25	0.95	1.90
OA[L2]	0.91	0.16	0.62	1.24
OA[L3]	0.49	0.09	0.33	0.67
Osteop	1.38	0.18	1.03	1.73
Osteop[L2]	0.86	0.11	0.64	1.07
Osteop[L3]	0.52	0.07	0.39	0.66
Gout	1.72	0.63	0.63	3.07
Gout[L2]	0.67	0.25	0.25	0.92
Gout[L3]	1.05	0.38	0.38	1.87
Periodontitis	1.96	0.28	1.43	2.51
Periodontitis[L2]	1.16	0.17	0.84	1.48
Periodontitis[L3]	0.80	0.11	0.59	1.03
Hearing	1.45	0.18	1.12	1.83
Hearing[L2]	0.97	0.12	0.75	1.23
Hearing[L3]	0.22	0.03	0.17	0.27
Hearing[L4]	0.26	0.03	0.20	0.33
Eye	1.59	0.20	1.25	2.01
Eye[L2]	0.97	0.12	0.12	1.27
Eye[L3]	0.10	0.01	0.08	0.14
Eye[L4]	0.22	0.03	0.18	0.28
Eye[L5]	0.30	0.04	0.24	0.38
Depression	1.08	0.23	0.62	1.54
Depression[L2]	0.64	0.14	0.37	0.91
Depression[L3]	0.44	0.09	0.25	0.63
Parkinson	2.62	1.30	0.14	5.31
Park[L2]	1.49	0.74	0.08	3.03
Park[L3]	1.13	0.56	0.06	2.28
CI	3.12	0.89	1.50	5.01
CI[L2]	2.12	0.61	1.02	3.41
CI[L3]	1.00	0.28	0.48	1.60
Cancer	2.84	0.40	2.05	3.63

Table S6B.Time-1 Single-System Models Predicting Longitudinal BODN in BLSA Data

Systems	Disease	Posterior Coef	SE	2.5QI	97.5QI
CVD	Hypertension (HTN)	1.09	0.13	0.84	1.34
	HTN[L2]	0.48	0.06	0.37	0.59
	HTN[L3]	0.61	0.07	0.47	0.75
	Ischemic Heart Disease (IHD)	0.92	0.20	0.54	1.31
	IHD[L2]	0.70	0.15	0.41	1.00
	IHD[L3]	0.22	0.05	0.13	0.31
	Congestive Heart Failure (CHF)	1.23	0.18	0.89	1.57
	CHF[L2]	0.41	0.06	0.29	0.52
	CHF[L3]	0.82	0.12	0.60	1.05
	Arrhythmia (Arr)	0.39	0.09	0.22	0.58
	Arr[L2]	0.07	0.02	0.04	0.10
	Arr[L3]	0.09	0.02	0.05	0.14
	Arr[L4]	0.09	0.02	0.05	0.13
	Arr[L5]	0.07	0.02	0.04	0.10
	Arr[L6]	0.07	0.02	0.04	0.10
	Pripheral Artery Disease	1.21	0.63	0.03	2.46
CVA	Stroke	1.61	0.23	1.17	2.07
	Stroke[L2]	0.68	0.10	0.49	0.87
	Stroke[L3]	0.18	0.03	0.13	0.23
	Stroke[L4]	0.76	0.11	0.55	0.97
Renal	Chronik Kidney Disease (CKD)	2.47	0.38	1.80	3.29
	CKD[L2]	0.96	0.15	0.70	1.28
	CKD[L3]	0.42	0.06	0.31	0.56
	CKD[L4]	0.37	0.06	0.27	0.49
	CKD[L5]	0.72	0.11	0.52	0.95
Metabolic	Diabetes Mellitus (DM)	1.04	0.19	0.71	1.43
	DM[L2]	0.19	0.03	0.13	0.26
	DM[L3]	0.07	0.01	0.05	0.10
	DM[L4]	0.28	0.05	0.19	0.39
	DM[L5]	0.12	0.02	0.09	0.17
	DM[L6]	0.36	0.07	0.25	0.50
	Lipid	0.17	0.19	-0.20	0.56
	Lipid[L2]	0.08	0.09	-0.09	0.26
	Lipid[L3]	0.09	0.10	-0.11	0.30
GILIV	Liver	1.28	0.26	0.85	1.87
	Liver[L2]	0.45	0.09	0.30	0.65
	Liver[L3]	0.49	0.10	0.32	0.71
	Liver[L4]	0.35	0.07	0.23	0.50
	Gastrointestinal disease (GID)	1.54	0.17	1.21	1.89
	GID[L2]	1.20	0.13	0.94	1.47
	GID[L3]	0.34	0.04	0.27	0.42
Respiratory	Chronic Obstructive Pulmonary Disease (COPD)	2.35	0.43	1.55	3.24
	COPD[L2]	1.76	0.32	1.16	2.43
	COPD[L3]	0.59	0.11	0.39	0.81
	Asthma	1.95	0.53	0.92	3.01
Dysthyroidism	Hypothroidism (Hypoth)	1.08	0.14	0.80	1.37
	Hypoth[L2]	0.67	0.09	0.50	0.85
	Hypoth[L3]	0.21	0.03	0.15	0.26
	Hypoth[L4]	0.21	0.03	0.15	0.26
	Hyperth	1.25	1.17	-1.04	3.60

Hematologic	Anemia	1.42	0.33	0.83	2.14
	Anemia[L2]	1.09	0.25	0.64	1.65
	Anemia[L3]	0.33	0.08	0.19	0.49
	Thrombocytopenia	0.69	0.62	-0.52	1.91
	WBC	1.61	0.72	0.26	3.00
	WBC[L2]	0.32	0.14	0.05	0.60
	WBC[L3]	1.29	0.58	0.21	2.40
MSK	Osteoartheritis (OA)	1.50	0.24	1.05	1.99
	OA[L2]	0.95	0.15	0.66	1.25
	OA[L3]	0.56	0.09	0.39	0.74
	Osteop	1.45	0.17	1.12	1.80
	Osteop[L2]	0.91	0.11	0.71	1.13
	Osteop[L3]	0.54	0.06	0.41	0.67
	Gout	1.52	0.54	0.61	2.70
	Gout[l2]	0.70	0.25	0.28	1.24
	Gout[l3]	0.82	0.29	0.33	1.46
Oral Health	Periodontitis	1.96	0.28	1.43	2.51
	Periodontitis[L2]	1.16	0.17	0.84	1.48
	Periodontitis[L3]	0.80	0.11	0.59	1.03
Sensory	Hearing	0.98	0.15	0.71	1.31
	Hearing[L2]	0.65	0.10	0.47	0.86
	Hearing[L3]	0.14	0.02	0.10	0.18
	Hearing[L4]	0.20	0.03	0.14	0.26
	Eye	1.35	0.18	1.06	1.74
	Eye[L2]	0.86	0.12	0.68	1.11
	Eye[L3]	0.08	0.01	0.06	0.10
	Eye[L4]	0.16	0.02	0.13	0.21
	Eye[L5]	0.24	0.03	0.19	0.31
CNS	Depression	1.03	0.23	0.57	1.48
	Depression[L2]	0.59	0.13	0.32	0.84
	Depression[L3]	0.44	0.10	0.25	0.64
	Parkinson (Park)	2.60	1.28	0.16	5.27
	Park[L2]	1.48	0.73	0.09	3.00
	Park[L3]	1.12	0.55	0.07	2.27
	Cognitive Impairment (CI)	2.88	0.84	1.32	4.63
	CI[L2]	2.02	0.59	0.92	3.24
	CI[L3]	0.86	0.25	0.40	1.39
Cancer	Cancer	2.84	0.40	2.05	3.63

Table S6C. Time-1 Disease Levels of All Organ Systems Predicting Longitudinal BODN in BLSA Data

Systems	Disease	Posterior Coef	SE	2.5QI	97.5QI
CVD	Hypertension (HTN)	0.48	0.06	0.36	0.61
	HTN[L2]	0.31	0.04	0.23	0.39
	HTN[L3]	0.17	0.02	0.13	0.22
	Ischemic Heart Disease (IHD)	0.23	0.11	0.02	0.44
	IHD[L2]	0.11	0.05	0.01	0.21
	IHD[L3]	0.12	0.06	0.01	0.23
	Congestive Heart Failure (CHF)	0.31	0.07	0.17	0.46
	CHF[L2]	0.22	0.05	0.12	0.33
	CHF[L3]	0.09	0.02	0.05	0.13
	Arrhythmia (Arr)	0.14	0.04	0.07	0.23
	Arr[L2]	0.05	0.02	0.03	0.09
	Arr[L3]	0.03	0.01	0.01	0.04
	Arr[L4]	0.02	0.01	0.01	0.03
	Arr[L5]	0.02	0.01	0.01	0.03
	Arr[L6]	0.02	0.01	0.01	0.03
	Pripheral Artery Disease	0.00	0.31	-0.61	0.61
CVA	Stroke	0.65	0.10	0.47	0.85
	Stroke[L2]	0.33	0.05	0.24	0.43
	Stroke[L3]	0.11	0.02	0.08	0.14
	Stroke[L4]	0.21	0.03	0.15	0.27
Renal	Chronik Kidney Disease (CKD)	0.89	0.14	0.65	1.21
	CKD[L2]	0.53	0.08	0.38	0.71
	CKD[L3]	0.07	0.01	0.05	0.10
	CKD[L4]	0.10	0.02	0.07	0.13
	CKD[L5]	0.20	0.03	0.15	0.28
Metabolic	Diabetes Mellitus (DM)	0.40	0.05	0.31	0.51
	DM[L2]	0.17	0.02	0.13	0.21
	DM[L3]	0.07	0.01	0.06	0.09
	DM[L4]	0.06	0.01	0.05	0.08
	DM[L5]	0.04	0.01	0.03	0.05
	DM[L6]	0.06	0.01	0.04	0.07
	Lipid	0.42	0.08	0.27	0.59
	Lipid[L2]	0.30	0.06	0.19	0.42
	Lipid[L3]	0.12	0.02	0.08	0.17
GILIV	Liver	0.81	0.14	0.57	1.12
	Liver[L2]	0.45	0.08	0.31	0.62
	Liver[L3]	0.13	0.02	0.09	0.18
	Liver[L4]	0.23	0.04	0.17	0.32
	Gastrointestinal disease (GID)	0.90	0.08	0.74	1.06
	GID[L2]	0.72	0.06	0.59	0.85
	GID[L3]	0.18	0.02	0.15	0.21
Respiratory	Chronic Obstructive Pulmonary Disease (COPD)	0.65	0.18	0.31	1.01
	COPD[L2]	0.51	0.14	0.24	0.79
	COPD[L3]	0.11	0.04	0.07	0.22
	Asthma	1.64	0.24	1.17	2.11

Dysthyroidism	Hypothroidism (Hypoth)	0.68	0.06	0.56	0.81
	Hypoth[L2]	0.49	0.04	0.40	0.58
	Hypoth[L3]	0.10	0.01	0.08	0.11
	Hypoth[L4]	0.10	0.01	0.08	0.11
	Hyperth	-0.03	0.52	-1.06	0.98
Hematologic	Anemia	0.81	0.18	0.50	1.21
	Anemia[L2]	0.56	0.12	0.35	0.83
	Anemia[L3]	0.25	0.06	0.16	0.38
	Thrombocytopenia	0.49	0.29	-0.07	1.05
	WBC	0.77	0.15	0.52	1.11
	WBC[L2]	0.18	0.03	0.12	0.26
	WBC[L3]	0.64	0.12	0.43	0.92
MSK	Osteoartheritis (OA)	0.84	0.11	0.64	1.07
	OA[L2]	0.59	0.08	0.45	0.75
	OA[L3]	2.52	0.03	0.19	0.32
	Osteop	0.52	0.07	0.39	0.66
	Osteop[L2]	0.42	0.06	0.32	0.53
	Osteop[L3]	0.10	0.01	0.07	0.13
	Gout	0.41	0.19	0.07	0.81
	Gout[L2]	0.23	0.11	0.04	0.46
	Gout[lL3]	0.18	0.08	0.03	0.35
Oral Health	Periodontitis	1.32	0.11	1.12	1.56
	Periodontitis[L2]	1.10	0.09	0.93	1.29
	Periodontitis[L3]	0.22	0.02	0.19	0.27
Sensory	Hearing	0.44	0.08	0.30	0.59
	Hearing[L2]	0.28	0.05	0.19	0.37
	Hearing[L3]	0.08	0.02	0.06	0.11
	Hearing[L4]	0.08	0.01	0.05	0.11
	Eye	0.67	0.10	0.50	0.88
	Eye[L2]	0.42	0.06	0.32	0.55
	Eye[L3]	0.05	0.01	0.04	0.07
	Eye[L4]	0.04	0.01	0.03	0.05
	Eye[L5]	0.15	0.02	0.11	0.19
CNS	Depression	0.71	0.10	0.51	0.91
	Depression[L2]	0.48	0.07	0.34	0.61
	Depression[L3]	23.43	0.03	0.17	0.30
	Parkinson (Park)	0.30	0.62	-1.06	1.47
	Park[L2]	0.14	0.30	-0.51	0.71
	Park[L3]	0.16	0.32	-0.55	0.76
	Cognitive Impairment (CI)	1.30	0.36	0.63	2.04
	CI[L2]	0.98	0.27	0.47	1.53
	CI[L3]	0.33	0.09	0.16	0.51
Cancer	Cancer	2.35	0.18	2.00	2.70

Table S6D. Time-2 Single Disease Models Predicting Longitudinal BODN in BLSA Data

Disease	Posterior Coef	SE	2.5QI	97.5QI
HTN	1.30	0.14	1.03	1.58
HTN[L2]	0.42	0.04	0.33	0.51
HTN[L3]	0.88	0.10	0.70	1.07
IHD	1.56	0.21	1.15	1.98
IHD[L2]	1.34	0.18	0.99	1.70
IHD[L3]	0.22	0.03	0.16	0.28
CHF	1.70	0.17	1.36	2.04
CHF[L2]	0.44	0.04	0.35	0.53
CHF[L3]	1.26	0.13	1.01	1.51
Arr	0.68	0.13	0.45	0.96
Arr[L2]	0.12	0.02	0.08	0.16
Arr[L3]	0.14	0.03	0.09	0.19
Arr[L4]	0.16	0.03	0.11	0.23
Arr[L5]	0.11	0.02	0.07	0.15
Arr[L6]	0.15	0.02	0.07	0.15
PAD	2.78	0.67	1.47	4.12
Stroke	1.59	0.22	1.17	2.02
Stroke[L2]	0.70	0.10	0.51	0.89
Stroke[L3]	0.16	0.02	0.12	0.20
Stroke[L4]	0.75	0.10	0.55	0.95
CKD	2.36	0.30	1.84	3.00
CKD[L2]	1.01	0.13	0.79	1.29
CKD[L3]	0.42	0.05	0.33	0.54
CKD[L4]	0.42	0.05	0.33	0.54
CKD[L5]	0.50	0.06	0.39	0.63
DM	0.88	0.12	0.66	1.12
DM[L2]	0.23	0.03	0.17	0.29
DM[L3]	0.06	0.01	0.05	0.08
DM[L4]	0.27	0.04	0.20	0.35
DM[L5]	0.13	0.02	0.10	0.17
DM[L6]	0.18	0.03	0.14	0.24
Lipid	0.55	0.18	0.19	0.91
Lipid[L2]	0.30	0.10	0.10	0.50
Lipid[L3]	0.25	0.08	0.09	0.41
Liver	1.33	0.18	1.02	1.72
Liver[L2]	0.60	0.08	0.46	0.77
Liver[L3]	0.53	0.07	0.41	0.69
Liver[L4]	0.20	0.03	0.15	0.26
GID	1.73	0.18	1.38	2.09
GID[L2]	1.37	0.14	1.09	1.65
GID[L3]	0.36	0.04	0.29	0.44
COPD	2.58	0.45	1.76	3.52
COPD[L2]	1.91	0.33	1.30	2.60
COPD[L3]	0.67	0.12	0.46	0.92
Asthma	2.45	0.54	1.39	3.53

Hypoth	1.15	0.14	0.88	1.44
Hypoth[L2]	0.53	0.06	0.40	0.66
Hypoth[L3]	0.30	0.04	0.23	0.37
Hypoth[L4]	0.32	0.04	0.25	0.40
Hyperth	1.89	1.44	-0.91	4.71
Anemia	1.51	0.31	0.95	2.18
Anemia[L2]	1.12	0.23	0.70	1.61
Anemia[L3]	0.39	0.08	0.25	0.57
Thrombocytopenia	1.55	0.64	0.32	2.79
WBC	0.66	0.39	-0.06	1.48
WBC[L2]	0.34	0.20	-0.03	0.77
WBC[L3]	0.32	0.19	-0.03	0.71
OA	1.28	0.22	0.89	1.74
OA[L2]	0.93	0.16	0.65	1.27
OA[L3]	0.35	0.06	0.24	0.47
Osteop	1.14	0.18	0.80	1.50
Osteop[L2]	0.81	0.13	0.57	1.07
Osteop[L3]	0.33	0.05	0.23	0.44
Periodontitis	1.73	0.25	1.26	2.25
Periodontitis[L2]	1.18	0.17	0.86	1.53
Periodontitis[L3]	0.55	0.08	0.40	0.72
Hearing	1.47	0.20	1.11	1.89
Hearing[L2]	0.90	0.12	0.68	1.15
Hearing[L3]	0.25	0.03	0.19	0.32
Hearing[L4]	0.31	0.04	0.23	0.40
Eye	1.57	0.18	1.25	1.96
Eye[L2]	0.99	0.11	0.79	1.23
Eye[L3]	0.08	0.01	0.06	0.10
Eye[L4]	0.25	0.03	0.20	0.31
Eye[L5]	0.25	0.03	0.20	0.31
Depression	1.42	0.26	0.92	1.93
Depression[L2]	0.74	0.14	0.48	1.00
Depression[L3]	0.68	0.12	0.44	0.93
Parkinson	2.43	0.74	0.99	3.89
Park[L2]	1.41	0.43	0.57	2.26
Park[L3]	1.02	0.31	0.42	1.63
CI	2.79	0.59	1.72	4.03
CI[L2]	2.01	0.42	1.24	2.90
CI[L3]	0.78	0.17	0.48	1.13
Cancer	2.85	0.40	2.07	3.63

Table S6E. Time-2 Single-System Models Predicting Longitudinal BODN in BLSA Data

Systems	Disease	Posterior Coef	SE	2.5QI	97.5QI
CVD	Hypertension (HTN)	0.97	0.14	0.71	1.25
	HTN[L2]	0.39	0.06	0.28	0.50
	HTN[L3]	0.58	0.08	0.43	0.75
	Ischemic Heart Disease (IHD)	0.98	0.21	0.58	1.39
	IHD[L2]	0.71	0.15	0.42	1.00
	IHD[L3]	0.27	0.06	0.16	0.39
	Congestive Heart Failure (CHF)	1.27	0.17	0.95	1.61
	CHF[L2]	0.32	0.04	0.24	0.40
	CHF[L3]	0.95	0.13	0.71	1.21
	Arrhythmia (Arr)	0.41	0.10	0.23	0.62
	Arr[L2]	0.07	0.02	0.04	0.11
	Arr[L3]	0.09	0.02	0.05	0.14
	Arr[L4]	0.09	0.02	0.05	0.14
	Arr[L5]	0.08	0.02	0.05	0.12
	Arr[L6]	0.07	0.02	0.04	0.11
	Pripheral Artery Disease	1.01	0.61	-0.19	2.19
CVA	Stroke	1.59	0.22	1.17	2.02
	Stroke[L2]	0.70	0.10	0.51	0.89
	Stroke[L3]	0.16	0.02	0.12	0.20
	Stroke[L4]	0.75	0.10	0.55	0.95
Renal	Chronik Kidney Disease (CKD)	2.36	0.30	1.84	3.00
	CKD[L2]	1.01	0.13	0.79	1.29
	CKD[L3]	0.42	0.05	0.33	0.54
	CKD[L4]	0.42	0.05	0.33	0.54
	CKD[L5]	0.50	0.06	0.39	0.63
Metabolic	Diabetes Mellitus (DM)	0.87	0.12	0.65	1.12
	DM[L2]	0.22	0.03	0.16	0.28
	DM[L3]	0.06	0.01	0.05	0.08
	DM[L4]	0.27	0.04	0.20	0.35
	DM[L5]	0.14	0.02	0.10	0.18
	DM[L6]	0.19	0.02	0.10	0.18
	Lipid	0.31	0.18	-0.04	0.65
	Lipid[L2]	0.17	0.10	-0.02	0.35
	Lipid[L3]	0.14	0.08	-0.02	0.30
GILIV	Liver	1.26	0.18	0.95	1.65
	Liver[L2]	0.49	0.07	0.37	0.64
	Liver[L3]	0.55	0.08	0.42	0.73
	Liver[L4]	0.21	0.03	0.16	0.28
	Gastrointestinal disease (GID)	1.63	0.17	1.29	1.96
	GID[L2]	1.30	0.14	1.03	1.57
	GID[L3]	0.33	0.03	0.26	0.39
espiratory	Chronic Obstructive Pulmonary Disease (COPD)	2.25	0.43	1.45	3.15
	COPD[L2]	1.71	0.33	1.10	2.39
	COPD[L3]	0.54	0.10	0.35	0.76

Re	Asthma	1.72	0.55	0.63	2.80
Dysthyroidism	Hypothyridism (Hypoth)	1.15	0.14	0.87	1.44
	Hypoth[L2]	0.54	0.07	0.41	0.68
	Hypoth[L3]	0.30	0.04	0.23	0.37
	Hypoth[L4]	0.31	0.04	0.23	0.39
	Hyperth	1.55	1.36	-1.14	4.18
Hematologic	Anemia	1.47	0.32	0.91	2.15
	Anemia[L2]	1.07	0.23	0.66	1.57
	Anemia[L3]	0.40	0.09	0.25	0.58
	Thrombocytopenia	1.29	0.62	0.08	2.51
	WBC	0.55	0.40	-0.19	1.40
	WBC[L2]	0.26	0.19	-0.09	0.66
	WBC[L3]	0.29	0.21	-0.10	0.74
MSK	Osteoartheritis (OA)	1.35	0.21	0.97	1.80
	OA[L2]	1.01	0.16	0.73	1.35
	OA[L3]	0.34	0.05	0.24	0.45
	Osteop	1.18	0.17	0.86	1.53
	Osteop[L2]	0.87	0.13	0.64	1.13
	Osteop[L3]	0.31	0.04	0.22	0.40
	Gout	1.48	0.39	0.83	2.37
	Gout[L2]	0.99	0.26	0.56	1.59
	Gout[L3]	0.49	0.13	0.27	0.78
Oral Health	Periodontitis	1.73	0.25	1.26	2.25
	Periodontitis[L2]	1.18	0.17	0.86	1.53
	Periodontitis[L3]	0.55	0.08	0.40	0.72
Sensory	Hearing	1.07	0.18	0.73	1.46
	Hearing[L2]	0.61	0.10	0.42	0.83
	Hearing[L3]	0.17	0.03	0.12	0.23
	Hearing[L4]	0.29	0.05	0.20	0.39
	Eye	1.39	0.17	1.09	1.76
	Eye[L2]	0.90	0.11	0.71	1.14
	Eye[L3]	0.07	0.01	0.05	0.09
	Eye[L4]	0.18	0.02	0.14	0.23
	Eye[L5]	0.22	0.03	0.17	0.28
CNS	Depression	1.28	0.25	0.80	1.78
	Depression[L2]	0.64	0.13	0.40	0.89
	Depression[L3]	0.64	0.13	0.40	0.89
	Parkinson (Park)	1.93	0.73	0.51	3.38
	Park[L2]	1.08	0.41	0.29	1.89
	Park[L3]	0.85	0.32	0.22	1.49
	Cognitive Impairment (CI)	2.37	0.58	1.30	3.58
	CI[L2]	1.66	0.41	0.91	2.51
	CI[L3]	0.71	0.17	0.39	1.07
Cancer	Cancer	2.85	0.40	2.07	3.63

Table S6F. Time-2 Disease Levels of All Organ Systems Predicting Longitudinal BODN in BLSA Data

Systems	Disease	Posterior Coef	SE	2.5QI	97.5QI
CVD	Hypertension (HTN)	0.38	0.06	0.26	0.49
	HTN[L2]	0.23	0.04	0.16	0.30
	HTN[L3]	0.15	0.02	0.10	0.19
	Ischemic Heart Disease (IHD)	0.19	0.10	0.01	0.38
	IHD[L2]	0.10	0.06	0.01	0.21
	IHD[L3]	0.09	0.05	0.00	0.17
	Congestive Heart Failure (CHF)	0.25	0.07	0.12	0.39
	CHF[L2]	0.15	0.04	0.07	0.23
	CHF[L3]	0.10	0.03	0.05	0.16
	Arrhythmia (Arr)	0.19	0.04	0.13	0.27
	Arr[L2]	0.10	0.02	0.07	0.14
	Arr[L3]	0.03	0.01	0.02	0.04
	Arr[L4]	0.02	0.00	0.01	0.03
	Arr[L5]	0.02	0.00	0.01	0.03
	Arr[L6]	0.03	0.01	0.02	0.04
	Pripheral Artery Disease	-0.04	0.27	-0.58	0.49
CVA	Stroke	0.72	0.09	0.56	0.89
	Stroke[L2]	0.40	0.05	0.31	0.49
	Stroke[L3]	0.13	0.02	0.10	0.16
	Stroke[L4]	0.19	0.02	0.15	0.24
Renal	Chronik Kidney Disease (CKD)	0.86	0.14	0.62	1.16
	CKD[L2]	0.52	0.08	0.37	0.70
	CKD[L3]	0.04	0.01	0.03	0.06
	CKD[L4]	0.07	0.01	0.05	0.09
	CKD[L5]	0.23	0.04	0.17	0.31
Metabolic	Diabetes Mellitus (DM)	0.37	0.04	0.30	0.45
	DM[L2]	0.21	0.02	0.17	0.26
	DM[L3]	0.04	0.00	0.04	0.05
	DM[L4]	0.04	0.00	0.03	0.05
	DM[L5]	0.04	0.00	0.04	0.05
	DM[L6]	0.03	0.00	0.03	0.04
	Lipid	0.50	0.07	0.38	0.64
	Lipid[L2]	0.45	0.06	0.34	0.57
	Lipid[L3]	0.06	0.01	0.04	0.07
GILIV	Liver	0.78	0.10	0.60	1.01
	Liver[L2]	0.46	0.06	0.35	0.60
	Liver[L3]	0.16	0.02	0.12	0.20
	Liver[L4]	0.16	0.02	0.12	0.20
	Gastrointestinal disease (GID)	0.94	0.08	0.79	1.09
	GID[L2]	0.79	0.07	0.66	0.92
	GID[L3]	0.15	0.01	0.13	0.17
spiratory	Chronic Obstructive Pulmonary Disease (COPD)	0.97	0.17	0.64	1.33
	COPD[L2]	0.78	0.14	0.51	1.06
	COPD[L3]	0.19	0.03	0.13	0.27

Body Clock	Res	Asthma	1.49	0.23	1.04	1.94
	Dysthyroidism	Hypothyridism (Hypoth)	0.76	0.06	0.64	0.87
		Hypoth[L2]	0.55	0.04	0.47	0.64
		Hypoth[L3]	0.11	0.01	0.10	0.13
		Hypoth[L4]	0.09	0.01	0.08	0.10
		Hyperth	1.32	0.55	0.24	2.40
	Hematologic	Anemia	0.77	0.11	0.56	1.01
		Anemia[L2]	0.65	0.09	0.47	0.85
		Anemia[L3]	0.12	0.02	0.09	0.16
		Thrombocytopenia	0.15	0.26	-0.37	0.66
		WBC	0.99	0.15	0.73	1.33
		WBC[L2]	0.76	0.12	0.56	1.02
		WBC[L3]	0.23	0.03	0.17	0.31
	MSK	Osteoartheritis (OA)	0.64	0.09	0.48	0.82
		OA[L2]	0.51	0.07	0.38	0.65
		OA[L3]	0.13	0.02	0.10	0.17
		Osteop	0.53	0.06	0.41	0.66
		Osteop[L2]	0.47	0.05	0.36	0.59
		Osteop[L3]	0.06	0.01	0.05	0.07
		Gout	0.21	0.18	-0.15	0.57
		Gout[l2]	0.11	0.10	-0.08	0.30
		Gout[l3]	0.10	0.08	-0.07	0.27
	Oral Health	Periodontitis	1.30	0.10	1.12	1.50
		Periodontitis[L2]	1.14	0.09	0.99	1.32
		Periodontitis[L3]	0.16	0.01	0.13	0.18
	Sensory	Hearing	0.39	0.08	0.24	0.56
		Hearing[L2]	0.21	0.04	0.13	0.31
		Hearing[L3]	0.07	0.01	0.04	0.10
		Hearing[L4]	0.11	0.02	0.07	0.16
		Eye	0.65	0.08	0.52	0.82
		Eye[L2]	0.47	0.06	0.37	0.59
		Eye[L3]	0.04	0.00	0.03	0.05
		Eye[L4]	0.04	0.00	0.03	0.05
		Eye[L5]	0.10	0.01	0.08	0.13
	CNS	Depression	0.96	0.11	0.76	1.17
		Depression[L2]	0.46	0.05	0.36	0.56
		Depression[L3]	0.50	0.06	0.40	0.61
		Parkinson (Park)	0.74	0.31	0.14	1.34
		Park[L2]	0.41	0.17	0.08	0.74
		Park[L3]	0.33	0.14	0.06	0.60
		Cognitive Impairment (CI)	1.22	0.25	0.76	1.76
		CI[L2]	0.83	0.17	0.52	1.20
		CI[L3]	0.39	0.08	0.24	0.56

	Cancer	Cancer	2.45	0.17	2.12	2.80

Table S6G. Single Disease Models Predicting Longitudinal BODN in InCHIANTI Data

Disease	Posterior Coef	SE	2.5QI	97.5QI
HTN	1.71	0.11	1.51	1.91
HTN[L2]	1.21	0.08	1.07	1.36
HTN[L3]	0.50	0.03	0.44	0.55
IHD	0.82	0.18	0.48	1.18
IHD[L2]	0.57	0.12	0.33	0.81
IHD[L3]	0.25	0.06	0.15	0.37
CHF	1.03	0.18	0.68	1.37
CHF[L2]	0.42	0.07	0.28	0.56
CHF[L3]	0.61	0.11	0.40	0.81
Arr	0.26	0.10	0.09	0.47
Arr[L2]	0.03	0.01	0.01	0.06
Arr[L3]	0.05	0.02	0.02	0.09
Arr[L4]	0.06	0.02	0.02	0.10
Arr[L5]	0.05	0.02	0.02	0.10
Arr[L6]	0.07	0.03	0.02	0.12
PAD	1.56	0.25	1.06	2.06
Stroke	0.83	0.15	0.54	1.12
Stroke[L2]	0.38	0.07	0.25	0.52
Stroke[L3]	0.23	0.04	0.15	0.31
Stroke[L4]	0.22	0.04	0.14	0.29
CKD	1.01	0.10	0.83	1.23
CKD[L2]	0.56	0.06	0.46	0.68
CKD[L3]	0.23	0.02	0.19	0.28
CKD[L4]	0.12	0.01	0.10	0.15
CKD[L5]	0.10	0.01	0.08	0.12
DM	0.54	0.15	0.31	0.89
DM[L2]	0.16	0.04	0.09	0.26
DM[L3]	0.11	0.03	0.06	0.18
DM[L4]	0.08	0.02	0.05	0.13
DM[L5]	0.09	0.03	0.05	0.15
DM[L6]	0.10	0.03	0.06	0.17
Lipid	0.74	0.10	0.55	0.94
Lipid[L2]	0.59	0.08	0.44	0.75
Lipid[L3]	0.15	0.02	0.11	0.19
Liver	0.70	0.15	0.43	1.00
Liver[L2]	0.28	0.06	0.17	0.40
Liver[L3]	0.29	0.06	0.18	0.41
Liver[L4]	0.13	0.03	0.08	0.19
GID	0.70	0.11	0.50	0.92
GID[L2]	0.52	0.08	0.37	0.68
GID[L3]	0.18	0.03	0.13	0.24
COPD	1.03	0.18	0.69	1.40
COPD[L2]	0.75	0.13	0.50	1.02
COPD[L3]	0.28	0.05	0.19	0.38
Asthma	1.85	0.24	1.38	2.33

Hypoth	0.97	0.26	0.47	1.50
Hypoth[L2]	0.41	0.11	0.20	0.63
Hypoth[L3]	0.29	0.08	0.14	0.45
Hypoth[L4]	0.27	0.07	0.13	0.42
Hyperth	2.34	0.27	1.81	2.88
Anemia	0.96	0.25	0.49	1.48
Anemia[L2]	0.65	0.17	0.33	1.01
Anemia[L3]	0.31	0.08	0.16	0.47
Thrombocytopenia	1.54	0.33	0.89	2.19
WBC	0.23	0.16	-0.08	0.54
WBC[L2]	0.12	0.08	-0.04	0.28
WBC[L3]	0.11	0.08	-0.04	0.26
OA	1.58	0.14	1.31	1.85
OA[L2]	1.12	0.10	0.93	1.31
OA[L3]	0.46	0.04	0.38	0.54
Osteop	0.48	0.23	0.02	0.94
Osteop[L2]	0.48	0.23	0.02	0.94
Osteop[L3]	0.00	0.00	0.00	0.00
Gout	0.82	0.29	0.33	1.46
Gout[L2]	0.43	0.15	0.17	0.77
Gout[L3]	0.39	0.14	0.16	0.69
Periodontitis	1.78	0.10	1.58	1.99
Periodontitis[L2]	1.26	0.07	1.12	1.41
Periodontitis[L3]	0.52	0.03	0.46	0.58
Hearing	1.04	0.20	0.69	1.44
Hearing[L2]	0.52	0.10	0.35	0.72
Hearing[L3]	0.26	0.05	0.17	0.36
Hearing[L4]	0.26	0.05	0.17	0.36
Eye	0.93	0.11	0.75	1.14
Eye[L2]	0.54	0.06	0.44	0.67
Eye[L3]	0.24	0.03	0.20	0.30
Eye[L4]	0.14	0.02	0.11	0.17
Eye[L5]	0.00	0.00	0.00	0.00
Depression	1.70	0.37	1.05	2.48
Depression[L2]	0.95	0.21	0.59	1.39
Depression[L3]	0.75	0.16	0.46	1.09
Parkinson	1.28	0.42	0.46	2.12
Park[L2]	0.67	0.22	0.24	1.10
Park[L3]	0.61	0.20	0.22	1.02
CI	1.47	0.23	1.08	1.95
CI[L2]	1.10	0.17	0.81	1.46
CI[L3]	0.37	0.06	0.27	0.49
Cancer	1.28	0.30	0.69	1.87

Table S6H. Single-System Models Predicting Longitudinal BODN in InCHIANTI Data

Systems	Disease	Posterior Coef	SE	2.5QI	97.5QI
CVD	Hypertension (HTN)	1.56	0.10	1.35	1.77
	HTN[L2]	1.18	0.07	1.02	1.34
	HTN[L3]	0.38	0.03	0.33	0.43
	Ischemic Heart Disease (IHD)	0.37	0.18	0.04	0.73
	IHD[L2]	0.28	0.13	0.03	0.55
	IHD[L3]	0.09	0.05	0.01	0.18
	Congestive Heart Failure (CHF)	1.20	0.16	0.21	1.48
	CHF[L2]	0.62	0.08	0.11	0.77
	CHF[L3]	0.58	0.08	0.10	0.71
	Arrhythmia (Arr)	0.30	0.08	0.18	0.30
	Arr[L2]	0.05	0.01	0.03	0.05
	Arr[L3]	0.06	0.01	0.03	0.06
	Arr[L4]	0.06	0.02	0.04	0.06
	Arr[L5]	0.06	0.01	0.04	0.06
	Arr[L6]	0.07	0.03	0.04	0.07
	Pripheral Artery Disease	0.80	0.23	0.35	1.26
CVA	Stroke	0.83	0.15	0.54	1.12
	Stroke[L2]	0.38	0.07	0.24	0.51
	Stroke[L3]	0.23	0.04	0.15	0.31
	Stroke[L4]	0.22	0.04	0.15	0.30
Renal	Chronik Kidney Disease (CKD)	2.02	0.10	0.84	2.22
	CKD[L2]	1.11	0.05	0.46	1.22
	CKD[L3]	0.46	0.02	0.19	0.51
	CKD[L4]	0.24	0.01	0.10	0.27
	CKD[L5]	0.21	0.02	0.09	0.22
Metabolic	Diabetes Mellitus (DM)	0.55	0.14	0.33	0.88
	DM[L2]	0.17	0.04	0.10	0.27
	DM[L3]	0.11	0.03	0.07	0.18
	DM[L4]	0.08	0.02	0.05	0.12
	DM[L5]	0.09	0.02	0.05	0.14
	DM[L6]	0.10	0.03	0.06	0.17
	Lipid	0.76	0.10	0.57	0.96
	Lipid[L2]	0.62	0.08	0.46	0.78
	Lipid[L3]	0.14	0.02	0.11	0.18
GILIV	Liver	0.72	0.14	0.46	1.01
	Liver[L2]	0.29	0.06	0.19	0.41
	Liver[L3]	0.29	0.06	0.19	0.41
	Liver[L4]	0.14	0.02	0.08	0.19
	Gastrointestinal disease (GID)	0.71	0.11	0.51	0.93
	GID[L2]	0.53	0.08	0.38	0.69
	GID[L3]	0.18	0.03	0.13	0.24
Respiratory	Chronic Obstructive Pulmonary Disease (COPD)	2.10	0.32	0.17	2.80
	COPD[L2]	1.40	0.21	0.11	1.87
	COPD[L3]	0.70	0.11	0.06	0.93
	Asthma	1.49	0.28	0.94	2.04

Dysthyroidism	Hypothroidism (Hypoth)	0.99	0.25	0.44	1.80
	Hypoth[L2]	0.41	0.10	0.18	0.74
	Hypoth[L3]	0.29	0.08	0.13	0.54
	Hypoth[L4]	0.29	0.07	0.13	0.52
	Hyperth	2.30	0.27	1.78	2.84
Hematologic	Anemia	1.20	0.24	0.44	1.80
	Anemia[L2]	0.84	0.16	0.30	1.26
	Anemia[L3]	0.36	0.08	0.14	0.54
	WBC	0.28	0.16	-0.03	0.59
	WBC[L2]	0.13	0.08	-0.01	0.28
	WBC[L3]	0.15	0.08	-0.02	0.31
	Thrombocytopenia	1.48	0.33	0.84	2.13
MSK	Osteoartheritis (OA)	1.62	0.13	1.36	1.88
	OA[L2]	1.18	0.09	0.99	1.37
	OA[L3]	0.44	0.04	0.37	0.51
	Osteoporosis	1.20	0.20	0.40	1.60
	Osteop[L2]	0.78	0.13	0.26	1.04
	Osteop[L3]	0.42	0.07	0.14	0.56
	Gout	0.86	0.22	0.49	1.36
	Gout[L2]	0.56	0.14	0.31	0.88
	Gout[L3]	0.30	0.08	0.18	0.48
Oral Health	Periodontitis	1.78	0.11	1.57	1.99
	Periodontitis[L2]	1.26	0.08	1.11	1.41
	Periodontitis[L3]	0.52	0.03	0.46	0.58
Sensory	Hearing	0.89	0.18	0.51	1.31
	Hearing[L2]	0.40	0.08	0.22	0.58
	Hearing[L3]	0.24	0.04	0.14	0.35
	Hearing[L4]	0.25	0.06	0.15	0.38
	Eye	1.10	0.09	0.67	1.50
	Eye[L2]	0.67	0.05	0.40	0.91
	Eye[L3]	0.28	0.02	0.17	0.38
	Eye[L4]	0.15	0.02	0.10	0.21
CNS	Depression	1.67	0.38	0.99	2.46
	Depression[L2]	0.83	0.19	0.49	1.23
	Depression[L3]	0.84	0.19	0.50	1.23
	Parkinson (Park)	1.02	0.39	0.26	1.78
	Park[L2]	0.52	0.19	0.13	0.90
	Park[L3]	0.50	0.20	0.13	0.88
	Cognitive Impairment (CI)	2.46	0.30	2.05	2.97
	CI[L2]	1.72	0.21	1.43	2.08
	CI[L3]	0.74	0.09	0.62	0.89
Cancer	Cancer	1.80	0.30	1.20	2.80

Table S6I. Disease Levels of All Organ Systems Predicting Longitudinal BODN in InCHIANTI Data

	System	Disease	Posteric	SE	2.5QI	97.5QI
Body Clock	CVD	Hypertension (HTN)	0.59	0.06	0.49	0.71
		HTN[L2]	0.51	0.05	0.42	0.62
		HTN[L3]	0.08	0.01	0.07	0.09
		Ischemic Heart Disease (IHD)	0.13	0.03	0.02	0.44
		IHD[L2]	0.06	0.01	0.01	0.21
		IHD[L3]	0.07	0.02	0.01	0.23
		Congestive Heart Failure (CHF)	0.30	0.07	0.12	0.39
		CHF[L2]	0.15	0.04	0.06	0.20
		CHF[L3]	0.15	0.04	0.06	0.20
		Arrhythmia (Arr)	0.10	0.04	0.01	0.14
		Arr[L2]	0.02	0.01	0.00	0.03
		Arr[L3]	0.02	0.01	0.00	0.02
		Arr[L4]	0.02	0.01	0.00	0.03
		Arr[L5]	0.02	0.01	0.00	0.03
		Arr[L6]	0.02	0.01	0.00	0.03
		Pripheral Artery Disease	0.08	0.31	0.12	0.32
	CVA	Stroke	0.55	0.07	0.27	0.71
		Stroke[L2]	0.21	0.03	0.10	0.27
		Stroke[L3]	0.18	0.02	0.09	0.23
		Stroke[L4]	0.17	0.02	0.08	0.21
	Renal	Chronik Kidney Disease (CKD)	0.60	0.05	0.10	0.91
		CKD[L2]	0.43	0.04	0.07	0.66
		CKD[L3]	0.07	0.01	0.01	0.10
		CKD[L4]	0.05	0.00	0.01	0.07
		CKD[L5]	0.05	0.00	0.01	0.08
	Metabolic	Diabetes Mellitus (DM)	0.30	0.06	0.20	0.41
		DM[L2]	0.11	0.02	0.07	0.15
		DM[L3]	0.06	0.01	0.04	0.09
		DM[L4]	0.04	0.01	0.02	0.05
		DM[L5]	0.04	0.01	0.03	0.05
		DM[L6]	0.05	0.01	0.03	0.07
		Lipid	0.43	0.05	0.34	0.52
		Lipid[L2]	0.36	0.04	0.28	0.43
		Lipid[L3]	0.07	0.01	0.06	0.09
	GILIV	Liver	0.62	0.08	0.22	0.95
		Liver[L2]	0.23	0.03	0.08	0.35
		Liver[L3]	0.23	0.03	0.08	0.35
		Liver[L4]	0.16	0.02	0.06	0.25
		Gastrointestinal disease (GID)	0.85	0.05	0.41	0.99
		GID[L2]	0.70	0.04	0.34	0.81
		GID[L3]	0.15	0.01	0.07	0.18
	Respiratory	Chronic Obstructive Pulmonary Disease (COPD)	0.42	0.09	0.30	0.81
		COPD[L2]	0.32	0.07	0.23	0.61
		COPD[L3]	0.11	0.02	0.08	0.20
		Asthma	0.70	0.13	0.45	0.94
	Dysthyroidism	Hypothroidism (Hypoth)	0.55	0.11	0.32	0.75
		Hypoth[L2]	0.30	0.06	0.17	0.41
		Hypoth[L3]	0.15	0.03	0.09	0.20
		Hypoth[L4]	0.10	0.02	0.06	0.14
		Hyperth	0.42	0.13	0.25	1.70

Hematologic	Anemia	0.31	0.11	0.01	0.84
	Anemia[L2]	0.20	0.07	0.01	0.56
	Anemia[L3]	0.11	0.04	0.00	0.29
	WBC	0.80	0.09	0.62	0.98
	WBC[L2]	0.26	0.03	0.20	0.31
	WBC[L3]	0.55	0.06	0.42	0.67
	Thrombocytopenia	0.32	0.12	-0.11	0.44
MSK	Osteoartheritis (OA)	0.70	0.07	0.49	0.93
	OA[L2]	0.53	0.05	0.38	0.70
	OA[L3]	0.17	0.02	0.12	0.22
	Osteoporosis	0.43	0.10	0.32	0.55
	Osteop[L2]	0.21	0.05	0.16	0.27
	Osteop[L3]	0.21	0.05	0.16	0.27
	Gout	0.48	0.11	0.32	0.72
	Gout[L2]	0.36	0.08	0.24	0.54
	Gout[L3]	0.12	0.03	0.08	0.18
Oral Health	Periodontitis	0.82	0.06	0.62	1.13
	Periodontitis[L2]	0.67	0.05	0.51	0.92
	Periodontitis[L3]	0.15	0.01	0.11	0.20
Sensory	Hearing	0.32	0.06	0.18	0.46
	Hearing[L2]	0.20	0.04	0.11	0.28
	Hearing[L3]	0.05	0.01	0.03	0.07
	Hearing[L4]	0.07	0.01	0.04	0.10
	Eye	0.52	0.05	0.22	0.80
	Eye[L2]	0.35	0.03	0.15	0.54
	Eye[L3]	0.06	0.01	0.02	0.09
	Eye[L4]	0.11	0.01	0.05	0.18
CNS	Depression	0.66	0.13	0.44	0.95
	Depression[L2]	0.46	0.09	0.30	0.66
	Depression[L3]	0.21	0.04	0.14	0.30
	Parkinson (Park)	0.50	0.21	0.12	0.88
	Park[L2]	0.25	0.10	0.06	0.43
	Park[L3]	0.26	0.11	0.06	0.45
	Cognitive Impairment (CI)	0.72	0.12	0.49	0.95
	CI[L2]	0.48	0.08	0.33	0.64
	CI[L3]	0.24	0.04	0.16	0.31
Cancer	Cancer	1.92	0.13	0.82	2.21

Table S7A. Single disease model weights in BLSA data

Dissease	Time-1Single Disease Weight	Time-2 Single Disease Weight
W_Age	90	96
W_Null Model	10	4
W_Age	87	94
W_IHD	13	6
W_Age	82	90
W_CHF	18	10
W_Age	89	95
W_Arrhythmia	11	5
W_Age	82	90
W_HTN	18	10
W_Age	89	96
W_PAD	11	4
W_Age	83	93
W_Stroke	17	7
W_Age	74	79
W_CKD	26	21
W_Age	82	88
W_DM	18	12
W_Age	89	96
W_Hyperlipidemia	11	4
W_Age	81	89
W_GID	19	11
W_Age	86	89
W_Liver	14	11
W_Age	87	94
W_COPD	13	6
W_Age	85	95
W_Asthma	15	5
W_Age	84	90
W_Hypoth	16	10
W_Age	90	97
W_Hyperth	10	3
W_Age	88	94
W_Anemia	12	6
W_Age	89	97
W_Thrombocytopenia	11	3
W_Age	89	96
W_WBC	11	4
W_Age	78	90
W_Periodontitis	22	10
W_Age	89	96
W_Osteoporosis	11	4
W_Age	84	93
W_Osteoartheritis	16	7
W_Age	89	95

W_Gout	11	5
W_Age	82	91
W_Hearing	18	9
W_Age	73	79
W_Eye	27	21
W_Age	86	93
W_Depression	14	7
W_Age	91	96
W_Parkinson	9	4
W_Age	91	96
W_Dementia	9	4
W_Age	86	93
W_Cancer	14	7

Table S7B. Single System Weights

System	Time-1SingleSystem Weight	Time-2SingleSystem Weight
W_Age	90	96
W_Null Model	10	4
W_Age	74	79
W_CVD	26	21
W_Age	83	86
W_Stroke	17	14
W_Age	75	80
W_Renal	25	20
W_Age	81	86
W_Metabolic	19	14
W_Age	76	80
W_GILIV	24	20
W_Age	83	92
W_Resp	17	8
W_Age	83	90
W_DisThyr	17	10
W_Age	87	92
W_Hematology	13	8
W_Age	81	90
W_Oral	19	10
W_Age	78	86
W_Msk	22	14
W_Age	67	71
W_Sensory	34	29
W_Age	86	91
W_CNS	14	9
W_Age	86	93
W_Cancer	14	7

Table S7C.Multisystem Model Weights in BLSA

Multisystem model(number of BODN)	Time-1 multisystem model weight	Time-2 multisystem model weight
W_Age	90	94
W_Null	10	4
W_Age	74	79
W_1	26	21
W_Age	69	76
W_2	31	24
W_Age	65	72
W_3	35	28
W_Age	51	56
W_4	49	44
W_Age	45	49
W_5	55	51
W_Age	41	41
W_6	59	59
W_Age	41	40
W_7	59	60
W_Age	33	32
W_8	67	68
W_Age	32	28
W_9	68	72
W_Age	27	22
W_10	73	78
W_Age	20	12
W_11	80	88
W_Age	20	12
W_12	80	88
W_Age	12	9
W_13	88	91

Table S7D. Model Weights in InCHIANTI data

System	SingleSystem weight	Multisystem model (BODN value)	Multisystem model weight
W_Age	93	W_Age	98
W_Null Model	7	W_Null	2
W_Age	74	W_Age	90
W_CVD	26	W_1	10
W_Age	85	W_Age	88
W_Stroke	15	W_2	12
W_Age	75	W_Age	70
W_Renal	25	W_3	30
W_Age	81	W_Age	60
W_Metabolic	19	W_4	40
W_Age	76	W_Age	55
W_GILIV	24	W_5	45
W_Age	80	W_Age	52
W_Resp	20	W_6	48
W_Age	81	W_Age	45
W_DisThyr	19	W_7	55
W_Age	85	W_Age	40
W_Hematology	15	W_8	60
W_Age	80	W_Age	35
W_Oral	20	W_9	65
W_Age	75	W_Age	30
W_Msk	25	W_10	70
W_Age	65	W_Age	22
W_Sensory	35	W_11	78
W_Age	85	W_Age	15
W_CNS	15	W_12	85
W_Age	89	W_Age	5
W_Cancer	11	W_13	95
